# Dual-Loaded Quercetin–Resveratrol Niosomes: Sustained In Vitro Release and Retained Bioactivity in Hummus During Simulated Gastrointestinal Digestion

**DOI:** 10.3390/ijms27198686

**Published:** 2026-09-28

**Authors:** Jade Alejandra Miramontes-Piedra, Miguel Ángel Robles-García, Alejandra Vázquez-Aguilar, Brenda Vega-Ruiz, Carmen Lizette Del-Toro-Sánchez, Fridha Viridiana Villalpando-Vargas, Sergio Yair Rodríguez-Preciado, Mariana Díaz-Zaragoza, Juan Carlos Barrera-de-León, Ernesto Ramírez-Briones, Ricardo Iván González-Vega

**Affiliations:** 1 Departamento de Ciencias de la Salud, Centro Universitario de los Valles (CUValles), Universidad de Guadalajara, Carretera a Guadalajara Km 45.5, Ameca 46600, Jalisco, Mexico; jade.miramontes4106@alumnos.udg.mx (J.A.M.-P.); brenda.vega@academicos.udg.mx (B.V.-R.); viridiana.villalpando@academicos.udg.mx (F.V.V.-V.); sergio.rodriguezp@academicos.udg.mx (S.Y.R.-P.); mariana.diaz@academicos.udg.mx (M.D.-Z.); juan.bdeleon@academicos.udg.mx (J.C.B.-d.-L.); 2 Departamento de Ciencias Médicas y de la Vida, Centro Universitario de la Ciénega (CUCIÉNEGA), Universidad de Guadalajara, Av. Universidad 1115, Lindavista, Ocotlán 47820, Jalisco, Mexico; 3 Departamento de Investigación y Posgrado en Alimentos (DIPA), Universidad de Sonora, Blvd. Luis Encinas y Rosales S/N, Col. Centro, Hermosillo 83000, Sonora, Mexico; alejandra.vazquez@academicos.udg.mx (A.V.-A.); carmen.deltoro@unison.mx (C.L.D.-T.-S.); 4 Departamento de Ecología Aplicada, Centro Universitario de Ciencias Biológicas y Agropecuarias (CUCBA), Universidad de Guadalajara, Camino Ing. Ramón Padilla Sánchez No. 2100, Las Agujas, Zapopan 45510, Jalisco, Mexico; ernesto.ramirez@academicos.udg.mx

**Keywords:** niosomes, quercetin–resveratrol, functional food

## Abstract

Quercetin (QUE) and resveratrol (RES) have complementary bioactivities but limited aqueous solubility and oral bioavailability. This study developed dual-loaded quercetin–resveratrol niosomes (QR-NIOs) for incorporation into hummus and screened quercetin:resveratrol molar ratios using antioxidant, erythroprotective, and enzyme-inhibition assays. The 50:50 formulation showed the broadest activity profile and produced vesicles with 90.63 ± 3.63% overall encapsulation efficiency, 149 ± 13 nm diameter, 0.21 ± 0.02 polydispersity index, and −34 ± 2 mV ζ-potential. After 24 h, dialysis passage was 51.63%, compared with 80.35% for the unencapsulated mixture. Following simulated intestinal digestion, enriched hummus yielded 5.8 milligrams of gallic acid equivalents per gram of dry weight (mg GAE/g D.W.) and 4.7 milligrams of quercetin equivalents per gram of dry weight (mg QE/g D.W.) in the dialyzable phenolic and flavonoid fractions, respectively, exceeding both controls while retaining antioxidant and enzyme-inhibitory activities. Over 28 days at 4 °C, microbial counts remained below detection limits and the tested pathogens were absent. Overall acceptability (8.23/9) did not differ significantly from commercial hummus. QR-NIO-enriched hummus represents a sensory-compatible functional-food prototype with sustained apparent release and increased intestinal recovery of assay-reactive phenolic and flavonoid compounds; compound-specific and in vivo validation remains necessary.

## 1. Introduction

Chronic noncommunicable diseases (NCDs), including obesity, type 2 diabetes mellitus, cardiovascular diseases, and neurodegenerative disorders, remain major causes of morbidity and mortality worldwide [1,2]. Despite their differences, these conditions are associated with oxidative stress, low-grade inflammation, mitochondrial dysfunction, and metabolic dysregulation [3,4]. Excessive reactive oxygen and nitrogen species can overwhelm antioxidant defenses, damage macromolecules, and activate nuclear factor kappa B (NF-κB) and the NOD-like receptor family pyrin domain-containing 3 (NLRP3) inflammasome [5]. Conversely, impaired nuclear factor erythroid 2-related factor 2 (Nrf2) signaling may weaken antioxidant responses and disrupt redox homeostasis [6]. These mechanisms have encouraged food-based strategies designed to preserve bioactive compounds during gastrointestinal transit.

The intestinal environment links diet with health through barrier function, microbial metabolism, immunity, endocrine signaling, and neural communication within the gut–microbiota–brain axis [7]. Dysbiosis and impaired barrier integrity may facilitate microbial-product translocation and systemic inflammatory signaling [8,9]. Dietary polyphenols and their metabolites have attracted attention as modulators of intestinal redox balance, microbial ecology, and barrier function [10]. Nevertheless, antioxidant or enzyme-inhibitory activity measured in vitro does not demonstrate microbiota modulation, epithelial transport, neuroprotection, or clinical efficacy. Digestive bioaccessibility must be distinguished from absorption, systemic bioavailability, and physiological effects.

Quercetin and resveratrol are polyphenols with complementary antioxidant, anti-inflammatory, and metabolic activities. Quercetin can scavenge reactive species and modulate Nrf2, NF-κB, and adenosine monophosphate (AMP)-activated protein kinase (AMPK), whereas resveratrol has been associated with sirtuin 1 activation, mitochondrial regulation, and inhibition of inflammatory mediators [11,12]. Their combination may broaden activity through complementary redox and molecular interactions. However, these interactions are not invariably synergistic; their magnitude and direction depend on the quercetin-to-resveratrol ratio, concentration, biological endpoint, environment, and delivery matrix [13]. The compounds exhibit limited aqueous solubility, chemical instability, extensive metabolism, and low oral bioavailability, restricting food incorporation and reducing availability during gastrointestinal transit [14,15].

Nano-enabled delivery systems can improve dispersibility, retention, and release of hydrophobic phytochemicals. Niosomes self-assemble from nonionic surfactants and cholesterol. Hydrophobic compounds can associate with the bilayer through hydrogen bonding, hydrophobic interactions, and van der Waals forces, while cholesterol influences bilayer packing, rigidity, and permeability [16]. Compared with phospholipid-based carriers, niosomes may offer chemical stability, formulation flexibility, and scalability for food applications [17]. Resveratrol-loaded niosomes and proniosomes have been investigated as functional-food ingredients, whereas quercetin and resveratrol have been coencapsulated in nanostructured carriers [18,19,20]. However, encapsulation efficiency and particle size alone cannot predict carrier performance. Gastrointestinal pH, ionic strength, enzymes, bile salts, and food constituents may alter vesicle organization and release. Liberation during simulated digestion indicates potential bioaccessibility rather than confirmed absorption or systemic bioavailability.

Hummus contains dietary fiber, plant protein, resistant starch, unsaturated lipids, and endogenous phytochemicals, making it a suitable matrix for polyphenol-loaded delivery systems [21]. Its semisolid structure may facilitate nanovesicle dispersion while providing a vehicle for ingredients. Nevertheless, the matrix may modify vesicle behavior, and enrichment must not compromise microbiological quality or sensory acceptance. To our knowledge, dual-loaded quercetin–resveratrol niosomes have not been incorporated into hummus and evaluated through an integrated assessment of ratio-dependent interactions, physicochemical properties, apparent release, simulated gastrointestinal digestion, biological activity, microbiological quality, and sensory acceptance.

The working hypothesis was that balanced co-loading of quercetin and resveratrol would provide an assay-dependent bioactivity profile and that niosomal incorporation into hummus would sustain apparent release and preserve activity during digestion without compromising microbiological or sensory quality. Accordingly, this study screened quercetin–resveratrol ratios, characterized dual-loaded niosomes, evaluated their in vitro release, and determined the effects of incorporation into hummus. The 50:50 formulation exhibited the broadest favorable bioactivity profile and produced homogeneous nanovesicles with high overall encapsulation efficiency and initial colloidal stability. Incorporation into hummus promoted digestion-dependent liberation of potentially bioaccessible phenolics while maintaining antioxidant and enzyme-inhibitory activities, microbiological quality, and sensory acceptance. These findings support QR-NIO-enriched hummus as a functional-food prototype while underscoring the need for compound-specific, transport, microbiome, safety, and in vivo studies before physiological or clinical benefits can be inferred.

## 2. Results

### 2.1. Selection of the Quercetin-to-Resveratrol Ratio

#### 2.1.1. Antioxidant Activity of Dual-Loaded Quercetin–Resveratrol Niosome (QR-NIO) Formulation

**Figure 1:**
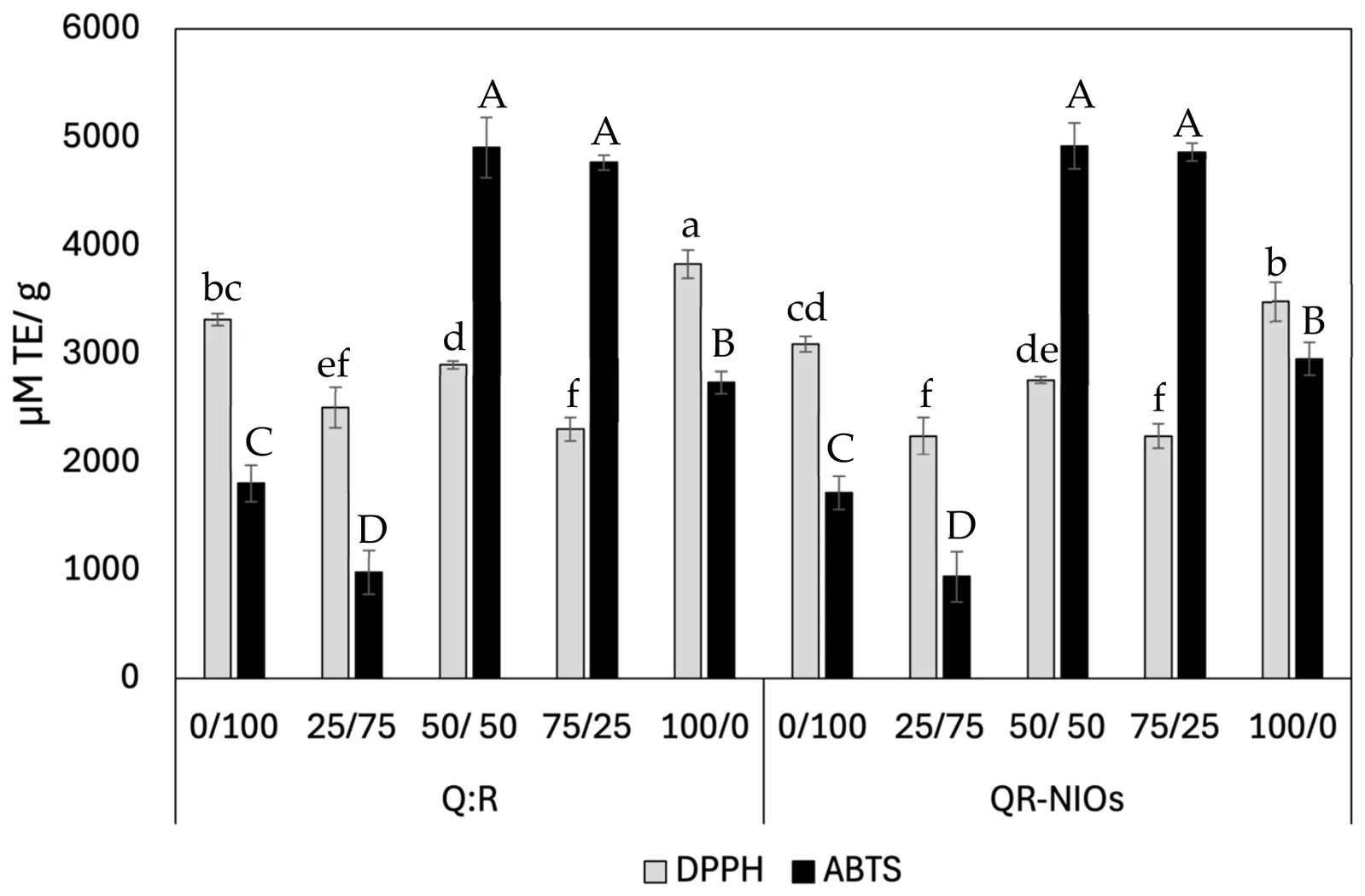
Antioxidant activity of free quercetin–resveratrol mixtures (Q:R) and their corresponding niosomal formulations (QR-NIOs), determined using the DPPH (gray bars) and ABTS (black bars) radical-scavenging assays. Quercetin:resveratrol (Q:R) ratios (*w*/*w*) were 0:100, 25:75, 50:50, 75:25, and 100:0; QR-NIOs denotes the niosomal formulations. Results are expressed as micromoles of Trolox equivalents per gram (µmol TE/g) and presented as mean ± standard deviation of three independent experiments (*n* = 3). DPPH and ABTS data were analyzed separately by two-way analysis of variance, with Q:R ratio and presentation (free or niosomal) as factors, followed by Tukey’s multiple-comparison test across the ten conditions within each assay (*p* < 0.05). Bars sharing at least one letter within the same assay do not differ significantly; bars with no letters in common differ significantly. Lowercase letters denote DPPH comparisons, and uppercase letters denote ABTS comparisons.

The antioxidant activity of free quercetin:resveratrol mixtures (Q:R) and their corresponding niosomal formulations (QR-NIOs) was evaluated using the 2,2-diphenyl-1-picrylhydrazyl (DPPH) and 2,2′-azino-bis(3-ethylbenzothiazoline-6-sulfonic acid) (ABTS) radical-scavenging assays across five quercetin (Q) ratios (Figure 1). In the DPPH assay, the 100:0 ratio showed the highest activity in both the free and niosomal groups (3833.45 ± 131.60 and 3479.95 ± 183.11 µmol TE/g D.W., respectively); these two values differed significantly. In contrast, ABTS activity was highest at the 50:50 and 75:25 ratios in both groups, with no significant difference between these ratios. The 25:75 ratio showed the lowest ABTS activity in both the free (983.60 ± 204.56 µmol TE/g D.W.) and niosomal (941.89 ± 235.41 µmol TE/g D.W.) groups. Separate two-way analyses of variance for each assay identified a significant effect of Q ratio on both DPPH and ABTS activity (*p* < 0.001). Presentation as a free mixture or niosomal formulation had a significant overall effect on DPPH activity (*p* < 0.001), but not on ABTS activity (*p* = 0.550); the interaction between ratio and presentation was not significant in either assay. Thus, the 50:50 and 75:25 ratios provided the strongest ABTS responses irrespective of presentation, whereas the 100:0 ratio yielded the highest DPPH response.

These findings are consistent with Lu et al. [22], who reported substantial DPPH-scavenging activity of quercetin and showed that niosomal encapsulation improved its solubility and photostability. Similarly, Machado et al. [23] found that resveratrol-loaded niosomes maintained DPPH activity comparable to that of free resveratrol, whereas ABTS activity depended on bilayer composition, with 96% retention in dodecanol-stabilized niosomes. In the present study, the similarly high ABTS values of the free and niosomal 50:50 and 75:25 mixtures indicate that their antioxidant activity was largely preserved after encapsulation. By contrast, the 100:0 ratio produced the highest DPPH activity in both presentations, with a significantly higher value for free quercetin than for its niosomal formulation. Together, these assay-dependent patterns show that antioxidant responses vary with the Q:R ratio and the assay conditions, without establishing a general increase in radical-scavenging activity following encapsulation.

#### 2.1.2. Protection of Human Erythrocytes Against Oxidative Damage Induced by 2,2′-Azobis(2-methylpropionamidine) Dihydrochloride (AAPH)

Erythroprotective activity differed significantly according to the quercetin-to-resveratrol ratio (Q:R) and ABO blood group and Rhesus D (RhD) antigen status (*p* < 0.001; Table 1). The 50:50 formulation produced the highest overall value in A RhD+ erythrocytes (100.45 ± 1.24%) and was also the most effective treatment in B RhD+ cells (87.54 ± 2.75%), significantly exceeding both single-compound formulations within these groups. In AB RhD+ erythrocytes, quercetin alone (100:0) provided the greatest protection (98.53 ± 2.75%), followed by resveratrol alone and the 50:50 formulation, which did not differ significantly from each other. Resveratrol alone (0:100) generated the highest response in O RhD+ cells (88.45 ± 1.67%), whereas quercetin alone showed the highest numerical value in O RhD− cells (81.87 ± 2.84%), without differing from the 75:25 formulation. Across groups, A RhD+ consistently showed high responses, while the 50:50 formulation displayed its lowest activity in O RhD− erythrocytes (64.73 ± 2.46%). Thus, formulation performance depended strongly on the specific blood-group phenotype.

Notably, the 25:75 formulation never produced the maximum response in any phenotype and yielded the lowest activity in A RhD+, B RhD+, and AB RhD+ erythrocytes. Conversely, the optimal treatment differed among groups, demonstrating a distinct formulation-by-phenotype response pattern and confirming that no quercetin–resveratrol ratio provided uniformly superior erythroprotection across samples. Overall, the 50:50 formulation performed best, achieving the highest erythroprotection and significantly surpassing both individual compounds in A RhD+ and B RhD+ erythrocytes.

Our blood-group-dependent pattern agrees with González-Vega et al. [24], who found that AAPH-induced erythroprotection by quercetin and antioxidants varied across ABO/RhD phenotypes. Likewise, Chen and Deuster [25] showed that quercetin markedly reduced AAPH-mediated hemolysis in human erythrocytes, supporting its membrane-protective contribution; however, neither study evaluated Q:R ratios or dual-loaded niosomes.

#### 2.1.3. Morphological Changes Induced by AAPH-Mediated Oxidative Stress

**Figure 2:**
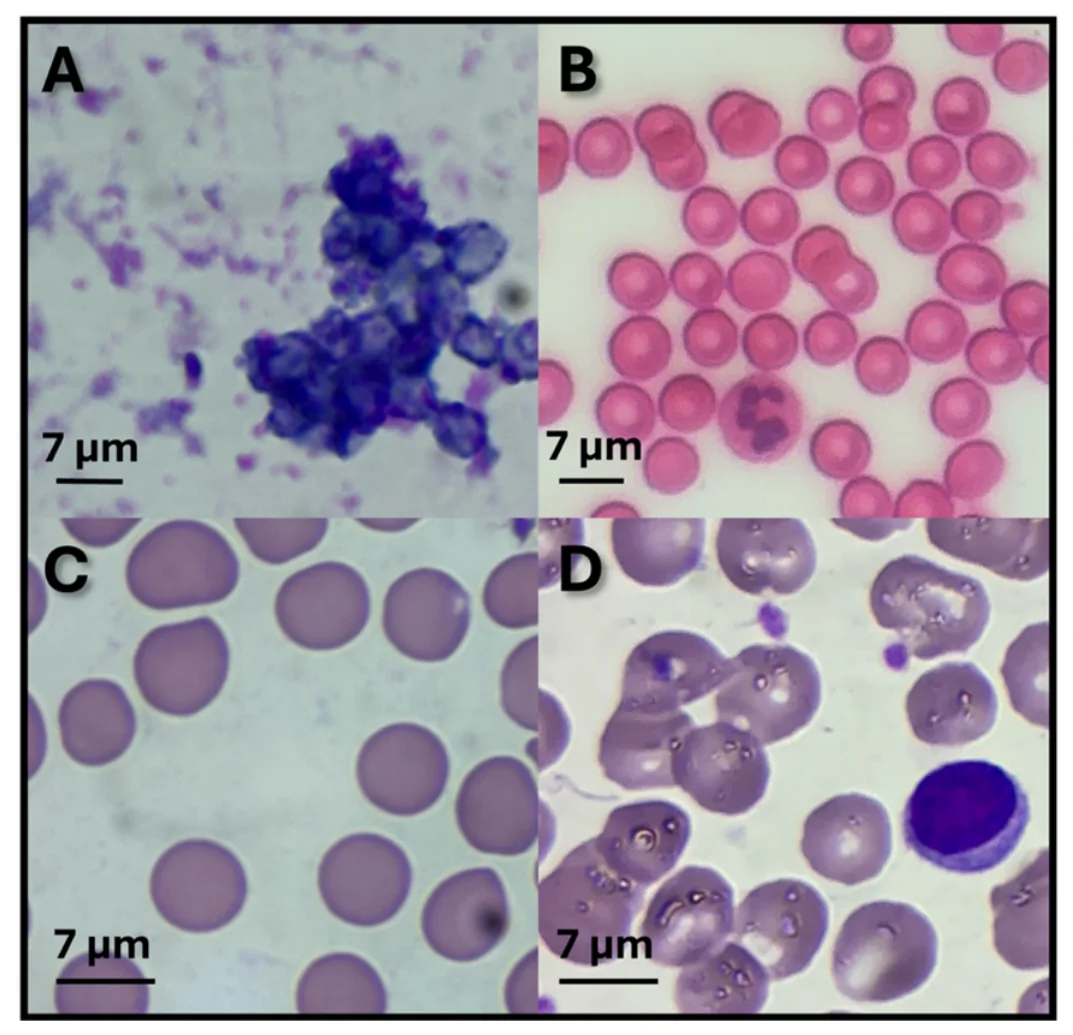
Morphological changes induced by AAPH-mediated oxidative stress and erythroprotective potential of QR-NIOs. (**A**) AAPH-induced hemolysis, positive control; (**B**) Erythrocytes without hemolysis, negative control; (**C**) AAPH-treated erythrocytes incubated with QR-NIOs (50/50), exhibiting 100.45 ± 1.24% (A RhD+) hemolysis inhibition; (**D**) Erythrocytes exposed to AAPH-induced oxidative stress and QR-NIOs (50/50), showing 64.73 ± 2.46 (O RhD−) inhibition of hemolysis.

Light microscopy corroborated the erythroprotective activity of QR-NIOs against AAPH-mediated oxidative damage (Figure 2). Erythrocytes exposed only to AAPH, the positive control (Figure 2A), showed extensive hemolysis, dense aggregates, indistinct membranes, and abundant debris. In contrast, erythrocytes without AAPH-induced hemolysis, the negative control (Figure 2B), retained uniform size, central pallor, regular contours, and discocyte morphology. AAPH-treated A RhD+ erythrocytes incubated with 50:50 QR-NIOs (Figure 2C) remained intact and morphologically homogeneous, closely resembling the negative control and corresponding to 100.45 ± 1.24% hemolysis inhibition. Under the same treatment, O RhD− erythrocytes (Figure 2D) exhibited heterogeneous staining, irregular contours, clear intracellular spaces, and occasional deformation, consistent with partial protection and 64.73 ± 2.46% inhibition. Therefore, the micrographs corroborated the blood-group-dependent efficacy quantified by the antihemolytic assay.

Collectively, antihemolytic measurements and microscopy demonstrated that 50:50 QR-NIOs provided maximal protection in A RhD+ erythrocyte but only partial protection in O RhD− cells. This phenotype-dependent preservation agrees with González-Vega et al. [24], who reported ABO/RhD-dependent antioxidant protection against AAPH-induced erythrocyte damage.

#### 2.1.4. Assessment of Porcine Pancreatic Elastase (PPE) Inhibition as an Indicator of Anti-Inflammatory Potential

**Figure 3:**
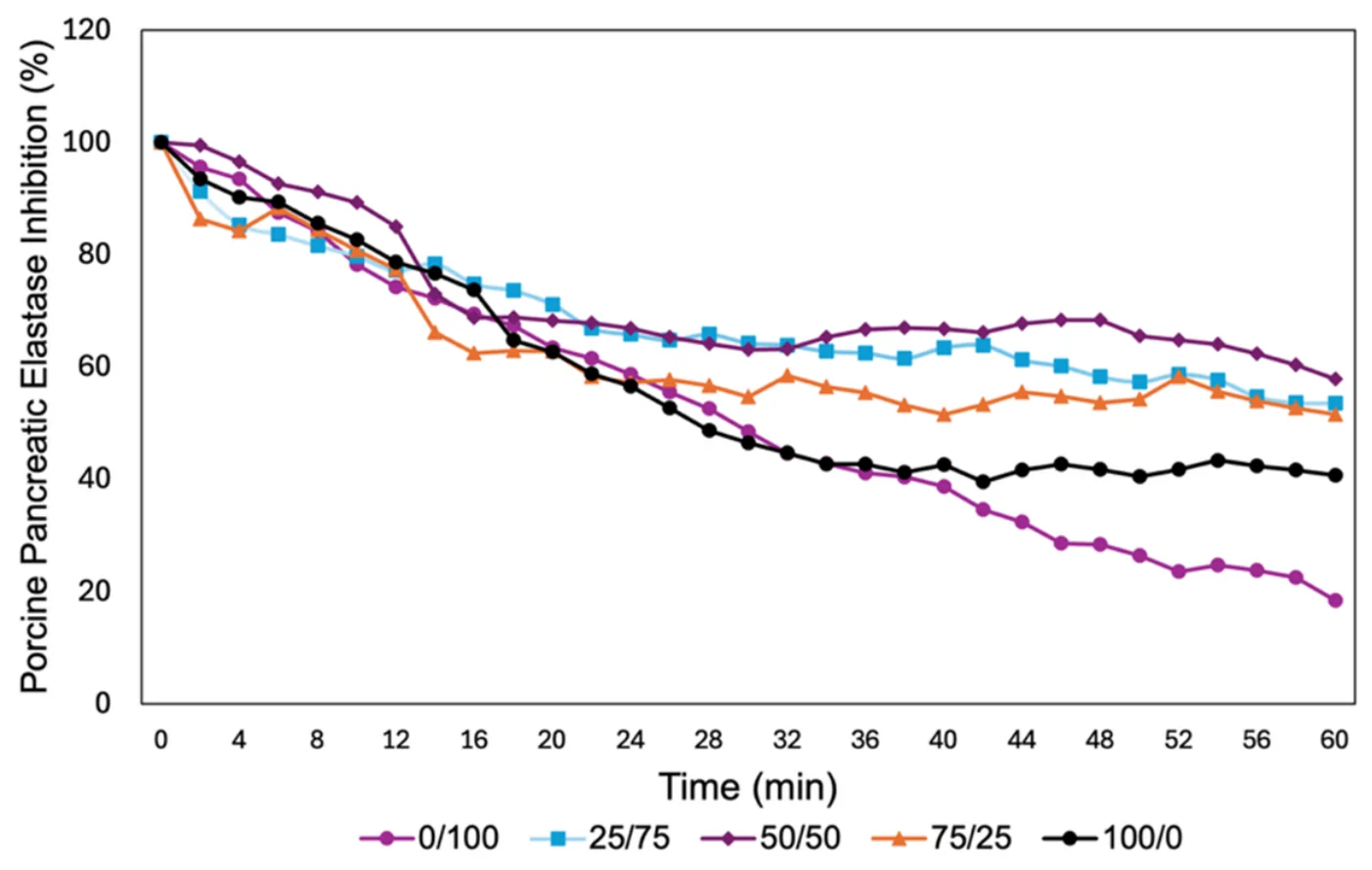
Time-dependent inhibition of porcine pancreatic elastase (PPE) by quercetin–resveratrol niosomal formulations with quercetin:resveratrol ratios of 0:100, 25:75, 50:50, 75:25, and 100:0 (*w*/*w*). PPE activity was monitored for 60 min using N-succinyl-Ala-Ala-Ala-p-nitroanilide as the substrate; substrate hydrolysis releases p-nitroaniline, so reduced product formation relative to the enzyme control indicates PPE inhibition. Inhibition values are plotted relative to the initial value of each formulation, set to 100% at 0 min.

Porcine pancreatic elastase (PPE) activity was assessed using N-succinyl-Ala-Ala-Ala-p-nitroanilide as the substrate (Figure 3). PPE-mediated hydrolysis releases p-nitroaniline; therefore, reduced p-nitroaniline formation relative to the enzyme control indicates inhibition of PPE activity. The 50:50 QR-NIO formulation showed the highest mean inhibition (57.86 ± 2.43%), followed by 25:75 (53.56 ± 1.36%), 75:25 (51.53 ± 2.63%), 100:0 (40.70 ± 1.35%), and 0:100 (18.42 ± 2.74%). The 50:50 formulation also showed the most persistent inhibition over the 60 min assay (Figure 3), supporting its selection for further evaluation. These findings are consistent with reports that polyphenols, including quercetin, can inhibit elastase activity [26,27]. However, the present assay does not establish the inhibition mechanism or demonstrate that co-loading directly prolongs a specific polyphenol–elastase interaction.

#### 2.1.5. Assessment of α-Amylase and α-Glucosidase Inhibitory Activity

**Figure 4:**
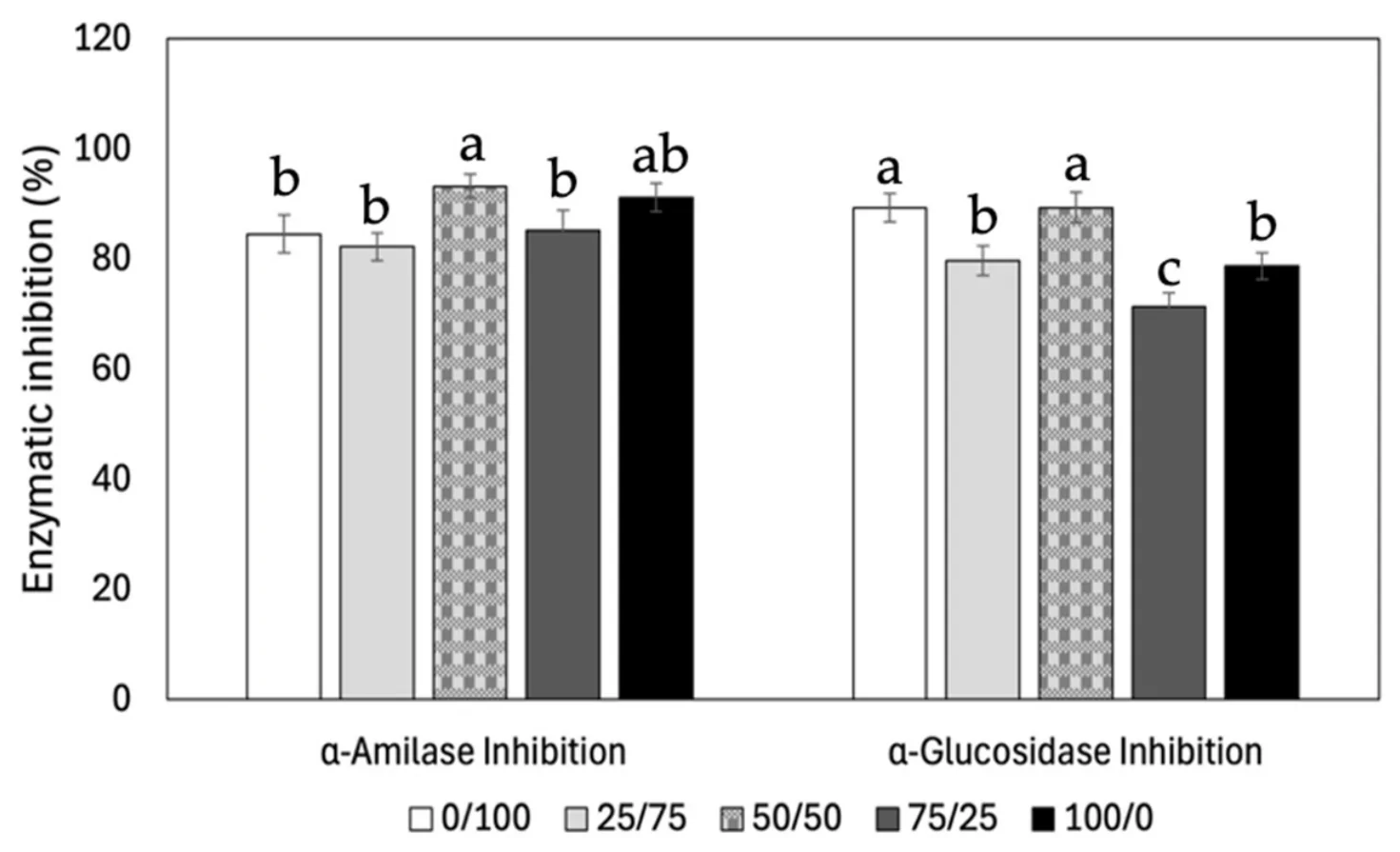
Inhibition of α-amylase and α-glucosidase by quercetin–resveratrol niosomal formulations with quercetin (Q) ratios of 0:100, 25:75, 50:50, 75:25, and 100:0 (*w*/*w*). Results are expressed as mean percentage inhibition ± standard deviation from three independent determinations (*n* = 3). Data for each enzyme were analyzed separately by one-way analysis of variance (ANOVA), followed by Tukey’s multiple-comparison test (*p* < 0.05). Within each enzyme assay, bars sharing at least one lowercase letter do not differ significantly; bars with no letters in common differ significantly. Letters must not be compared between the two enzyme assays.

α-Amylase inhibition differed according to the quercetin–resveratrol ratio (Figure 4). The 50:50 QR-NIO formulation showed the highest mean inhibition (93.23 ± 2.18%) and was assigned to statistical group a. This response was significantly greater than those of the 0:100, 25:75, and 75:25 formulations, whose inhibition values ranged from approximately 82% to 85% and were assigned to statistical group b. Quercetin-only niosomes (100:0) showed high activity, approaching 91.24 ± 2.56%, but their assignment to group ab indicated no significant difference from either 50:50 or the lower formulations. Consequently, the balanced 50:50 formulation provided the clearest enhancement of α-amylase inhibition, although its activity did not significantly exceed quercetin alone under these assay conditions.

**Figure 5:**
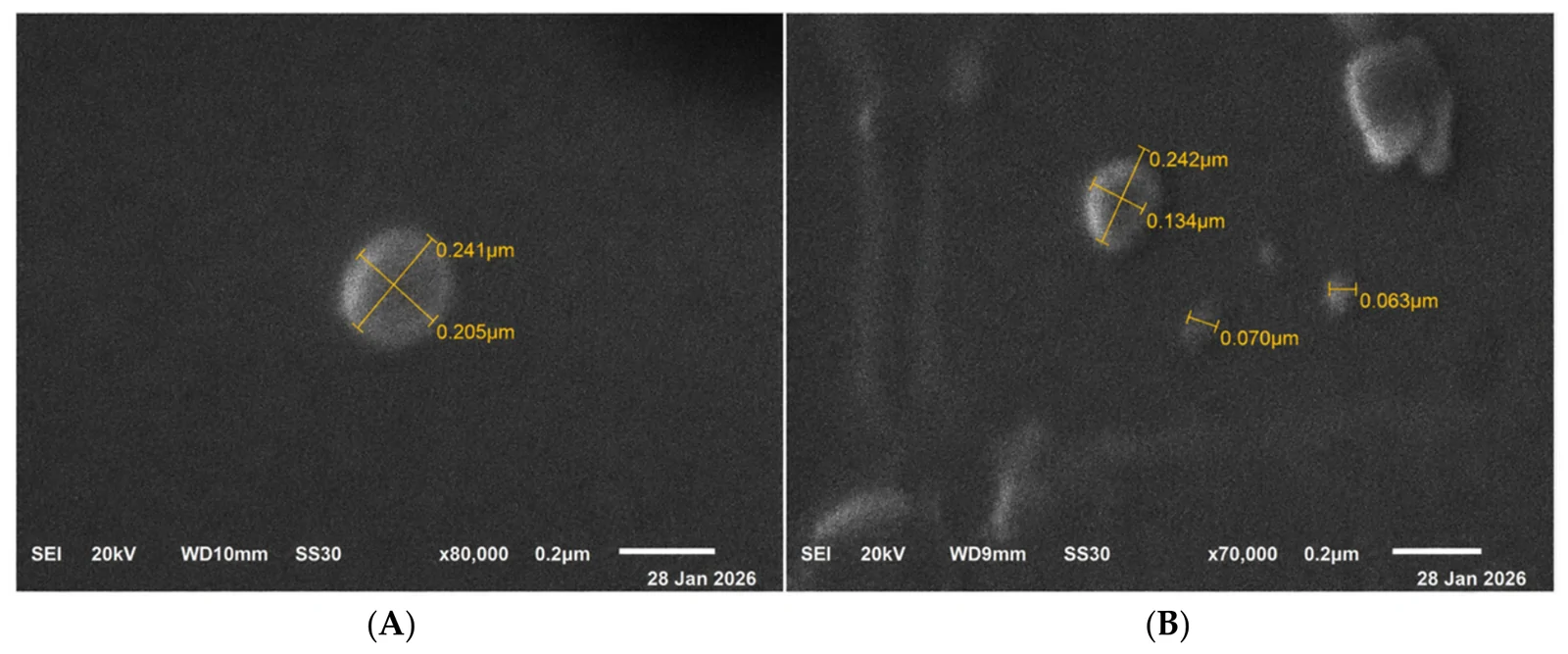
Morphology characterization of dual-loaded quercetin–resveratrol niosomes (QR-NIOs) observed by scanning electron microscopy (SEM). (**A**) Magnification: ×80,000; scale bar = 0.2 µm. (**B**) Magnification: ×70,000; scale bar = 0.2 µm.

α-Glucosidase inhibition depended significantly on the quercetin–resveratrol ratio (Figure 5), but the response pattern differed from that observed for α-amylase. Resveratrol-only niosomes (0:100) and the balanced 50:50 formulation produced the highest activities, both reaching 88.63 ± 3.85% inhibition and sharing group a. The 25:75 and quercetin-only 100:0 formulations showed intermediate responses of 78 ± 2.68% and were assigned to group b, demonstrating significantly lower inhibition than the two leading formulations. The 75:25 formulation exhibited the weakest activity (71 ± 3.62%) and formed an independent statistical group c, differing significantly from all others. Thus, increasing the quercetin proportion did not progressively enhance α-glucosidase inhibition. Instead, maximal activity was retained when resveratrol was used alone or combined equally with quercetin. Across both enzyme assays, 50:50 was the only formulation positioned within the highest statistical group for α-amylase and α-glucosidase, identifying it as the most consistently active ratio against sequential carbohydrate-hydrolyzing enzymes under the tested conditions.

The dual-enzyme response of 50:50 QR-NIOs agrees with Wang et al. [28], whose slow-release quercetin emulsion inhibited α-amylase and α-glucosidase and delayed starch hydrolysis, supporting the importance of delivery systems for preserving polyphenol activity. Zhang et al. [29] likewise demonstrated that resveratrol inhibited mammalian intestinal α-glucosidase and reduced postprandial glycemia in mice. These studies support the contributions of quercetin to α-amylase inhibition and resveratrol to α-glucosidase inhibition observed here. However, because 50:50 did not significantly outperform the best single-compound formulation in either assay, its broader dual-enzyme activity should be interpreted as complementary rather than synergistic.

#### 2.1.6. Analysis of Interactions Between QR-NIOs

Interactions between quercetin and resveratrol were evaluated by comparing observed activity with an additive response calculated from each compound’s proportional contribution. The interaction index (II), defined as experimental activity divided by theoretical activity, classified formulations as synergistic (II > 1), additive (II ≈ 1), or antagonistic (II < 1), thereby identifying combinations with effects exceeding those predicted from individual activities.

##### Antioxidant Interactions

DPPH scavenging varied modestly with the quercetin–resveratrol ratio, while the interaction analysis showed predominantly additive behavior (Table 2). Resveratrol alone (0:100) produced 49.27% inhibition, whereas quercetin alone (100:0) reached 56.89%, confirming the greater activity of quercetin under these assay conditions. Among dual-loaded formulations, 50:50 showed the highest experimental inhibition (53.41%), followed by 75:25 (51.74%) and 25:75 (51.33%). For 25:75 and 50:50, the experimental values closely matched their expected activities (51.18% and 53.08%), generating interaction indices of 1.003 and 1.006, respectively. These deviations of less than 1% support essentially additive effects rather than meaningful synergism. Conversely, the 75:25 formulation showed lower experimental activity than expected (51.74% versus 54.99%), resulting in an index of 0.941 and indicating a modest antagonistic deviation. Therefore, increasing the quercetin proportion above 50% did not proportionally enhance scavenging, and none of the mixtures exceeded the antioxidant activity of quercetin alone within the conditions evaluated in this study.

Meanwhile, the ABTS assay revealed ratio-dependent interactions between quercetin and resveratrol (Table 3). The 50:50 formulation produced the highest experimental activity (95.46%) and interaction index (II = 1.123), demonstrating synergism. The 75:25 formulation was also synergistic (92.76%; II = 1.083). Conversely, 25:75 showed the lowest activity (73.83%) and an antagonistic interaction (II = 0.876), confirming that antioxidant cooperation required balanced or quercetin-rich proportions.

The interaction analysis shown in Table 4 confirmed that erythroprotection depended on the quercetin–resveratrol ratio and phenotype. The 50:50 formulation was synergistic in A RhD+ (II = 1.104) and B RhD+ (II = 1.200), but additive in AB RhD+ (II = 0.969) and antagonistic in O RhD+ (II = 0.929) and O RhD− (II = 0.818). This pattern agrees with González-Vega et al. [24], who reported ABO/RhD-dependent protection against AAPH-generated radicals. Differences in membrane organization may influence polyphenol–membrane interactions, although this mechanism was not tested. The 25:75 formulation was antagonistic in A, B, AB, and O RhD− erythrocytes, whereas 75:25 produced only mild synergy in B RhD+, emphasizing the narrow phenotype-specific range of favorable interactions. Thus, quercetin–resveratrol synergy was strongest in A and B erythrocytes, while O phenotypes did not benefit from the equimolar combination.

##### Interactions in Porcine Pancreatic Elastase Inhibition

All three mixed QR-NIO formulations produced greater porcine pancreatic elastase (PPE) inhibition than their theoretical additive expectations under the tested conditions (Table 5). The observed and expected inhibition values were 53.56% and 23.99% for 25:75, 57.86% and 29.56% for 50:50, and 51.53% and 35.13% for 75:25, yielding interaction indices (II) of 2.233, 1.957, and 1.467, respectively. Thus, all three mixtures met the predefined criterion for apparent synergy (II > 1.05). The 50:50 formulation produced the highest observed PPE inhibition, whereas 25:75 showed the greatest proportional increase over its additive expectation. These indices describe the responses at the concentrations tested and do not establish synergy across a dose–response range.

##### Interactions in α-Amylase and α-Glucosidase Inhibition

According to Table 6, α-amylase inhibition and the interaction index varied with the quercetin-to-resveratrol ratio. The 50:50 formulation showed the highest observed inhibition (93.23%), exceeding its expected additive activity of 87.91% (II = 1.061). It was the only mixture classified as apparently synergistic under the predefined criterion (II > 1.05). The 25:75 and 75:25 formulations showed inhibition of 82.23% and 85.24%, respectively, slightly below their expected activities of 86.24% and 89.57%. Their interaction indices (0.954 and 0.952, respectively) fell within the predefined additive range. Among the single-compound formulations, quercetin (100:0) showed higher inhibition than resveratrol (0:100; 91.24% versus 84.57%). These classifications describe deviations from the calculated additive expectation at the tested concentrations and do not, by themselves, establish statistically significant interactions.

α-Glucosidase inhibition varied with the quercetin–resveratrol ratio (Table 7). The 50:50 formulation matched resveratrol alone as the highest experimental response (89.34%) and exceeded its additive expectation (84.04%; II = 1.063), demonstrating synergism. Conversely, 25:75 and 75:25 produced 79.74% and 71.42% inhibition, respectively, with antagonistic indices of 0.920 and 0.878. Thus, equal co-loading provided the most favorable combined enzymatic response overall.

### 2.2. Characterization and Evaluation of the Selected 50:50 QR-NIO Formulation

#### 2.2.1. Scanning Electron Microscopy (SEM)

Niosomes are vesicular nanocarriers composed of nonionic surfactants that typically exhibit spherical or ovoid morphology when assembled [30]. SEM revealed rounded-to-ellipsoidal QR-NIOs with nanometric dimensions (Figure 5). Panel A showed a particle measuring approximately 241 × 205 nm, whereas Panel B displayed an elongated particle of 242 × 134 nm and smaller structures measuring 63–70 nm, demonstrating clear dimensional heterogeneity.

The relatively smooth surfaces and limited aggregation support the formation of discrete nanoscale particles, whereas flattening and elongation may reflect dehydration and vacuum exposure during SEM preparation. The principal innovation is the development of a dual-loaded quercetin–resveratrol niosomal system with preserved nanoscale morphology; however, SEM alone cannot conclusively confirm co-encapsulation, which requires compound-specific encapsulation efficiencies and complementary independent spectroscopic evidence.

#### 2.2.2. Physicochemical Characterization of Colloidal Stability

Physicochemical characterization demonstrated that QR-NIOs formed a relatively homogeneous nanoscale dispersion, characterized by nanometric size, narrow size distribution, high encapsulation efficiency, and sufficient surface charge to support initial colloidal stability [30]. The co-formulated niosomes had a reported overall encapsulation efficiency of 90.63 ± 3.63%. In separate single-compound preparations, the measured vesicle-associated concentrations were 1.863 mM for quercetin and 1.7092 mM for resveratrol, from an initial concentration of 2 mM each. Across three independent preparations, the mean encapsulation efficiencies were 93.35 ± 2.31% for quercetin and 85.64 ± 2.11% for resveratrol. The mean hydrodynamic diameter of 149 ± 13.32 nm confirmed their nanometric size. The low polydispersity index (0.21 ± 0.02) demonstrated a comparatively narrow size distribution, and the negative ζ-potential (−34 ± 2.0 mV) suggested sufficient electrostatic repulsion to limit vesicle aggregation. These parameters support the initial colloidal stability and suitability of QR-NIOs for incorporation into functional food matrices, although stability during storage and gastrointestinal digestion remains to be experimentally confirmed.

**Figure 6:**
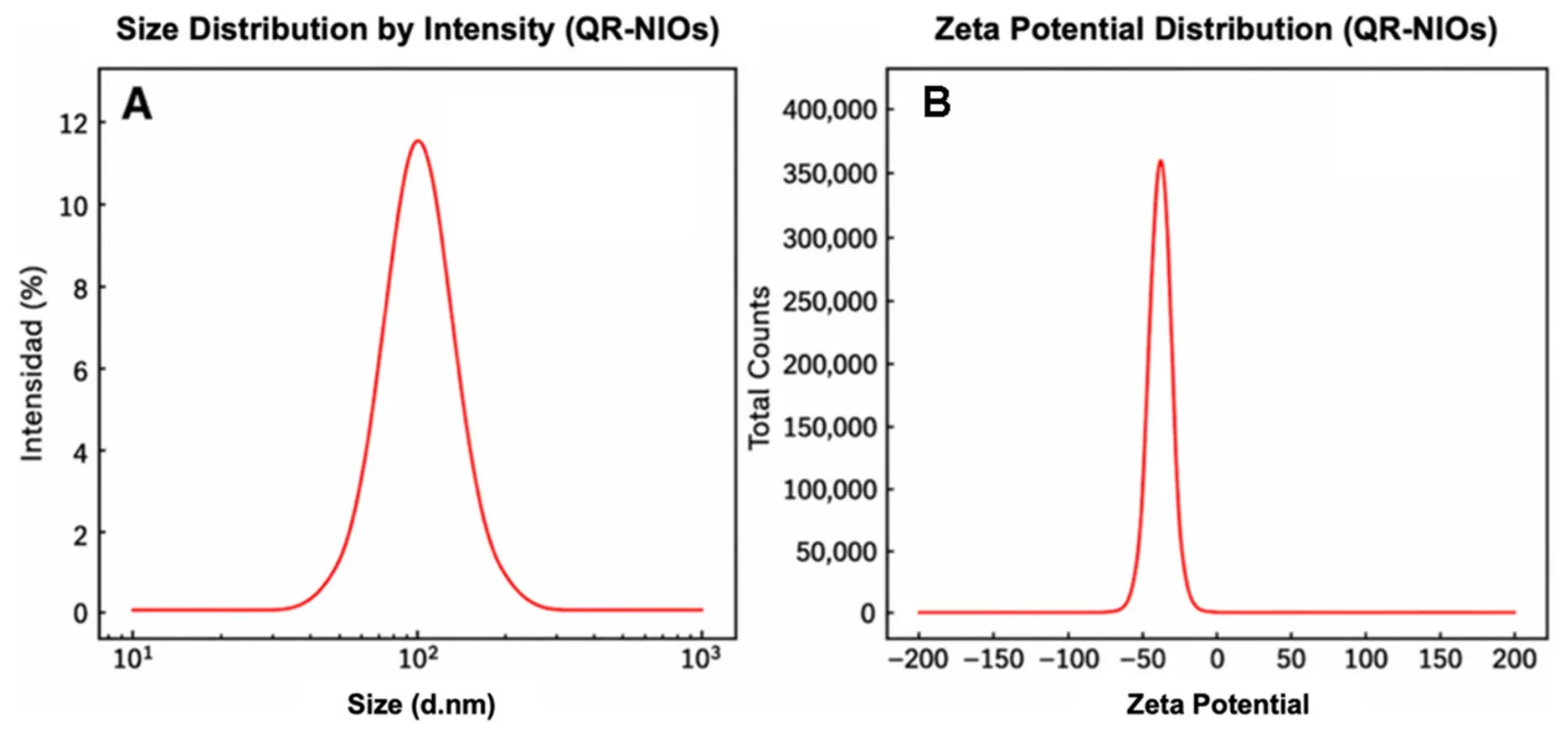
Particle-size distribution by intensity (**A**) and ζ-potential distribution (**B**) of the selected synergistic dual-loaded quercetin–resveratrol niosomes (QR-NIOs), determined by dynamic light scattering and electrophoretic light scattering, respectively.

The intensity-weighted distribution displayed a single dominant nanometric population without clearly resolved secondary peaks (Figure 6A), supporting limited aggregation and agreement with the reported particle size and polydispersity index (PDI). The difference between the modal peak and the mean diameter may arise from intensity weighting and the cumulant analysis used by dynamic light scattering (DLS). Likewise, the narrow negative ζ-potential peak (Figure 6B) indicated similar interfacial charge among most vesicles and was broadly consistent with the reported mean value. Although the combined distributions demonstrate a uniform initial formulation, they do not establish long-term stability under the ionic strength, pH, protein interactions, or bile-salt exposure expected in hummus and gastrointestinal environments.

#### 2.2.3. In Vitro Release and Kinetic Modeling

**Figure 7:**
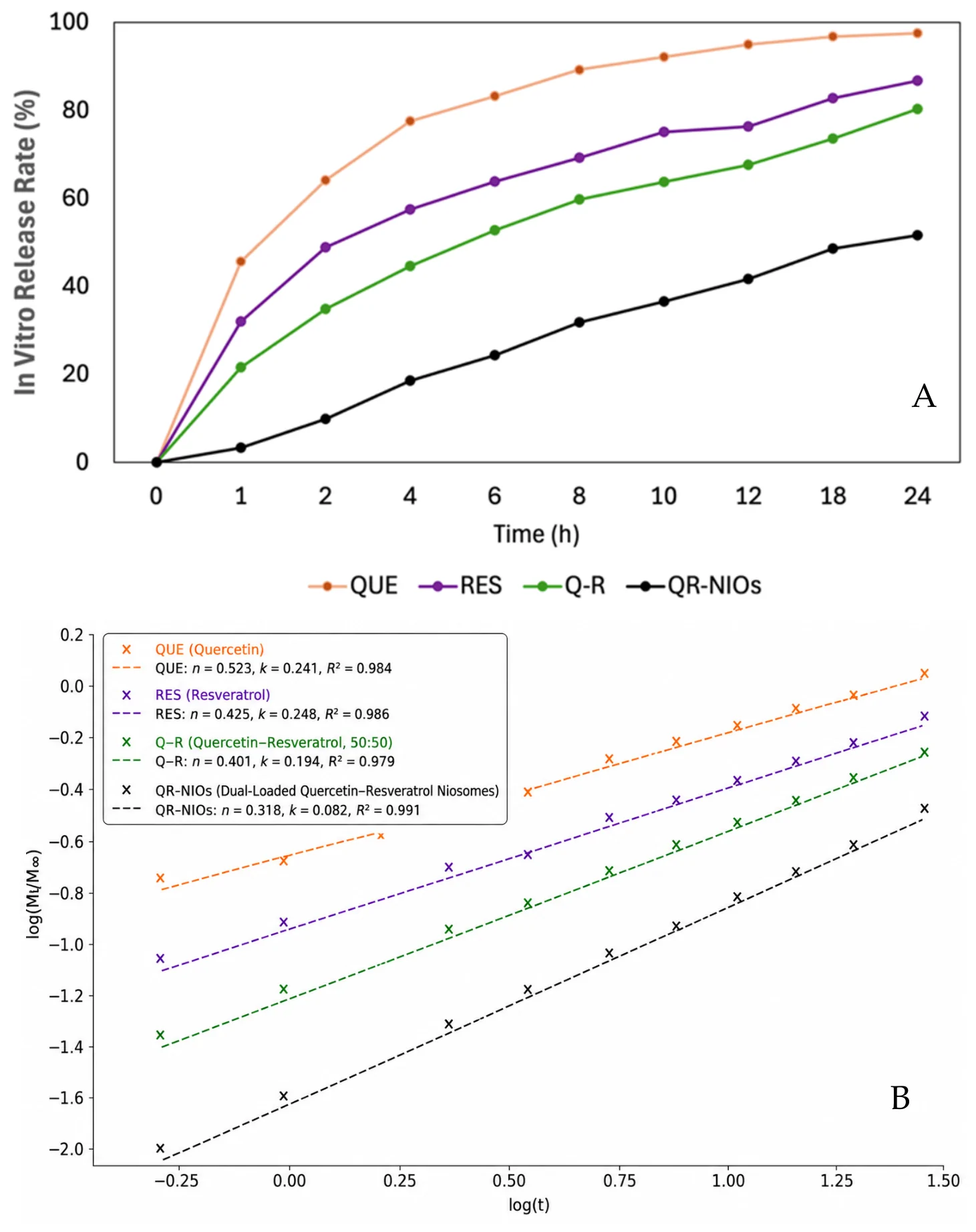
In vitro release and kinetic behavior of quercetin–resveratrol formulations. (**A**) Cumulative release profiles of free quercetin (QUE), free resveratrol (RES), their unencapsulated 50:50 quercetin-resveratrol (Q–R) mixture, and dual-loaded quercetin–resveratrol niosomes (QR-NIOs) in phosphate-buffered saline (PBS; pH 7.4) through a dialysis membrane (<12 kDa) at 37 °C. (**B**) Korsmeyer–Peppas model fitted to the corresponding release profiles. Symbols represent experimental data, while dashed lines indicate the fitted models and their respective kinetic parameters (n, k, and R^2^; see Tables S1 and S2 in the Supplementary Materials).

The release profiles differed markedly among formulations (Figure 7A, Table S1). Free QUE diffused fastest, increasing from 45.69% at 1 h to 97.52% at 24 h, followed by RES (32.06–86.74%) and the unencapsulated quercetin–resveratrol (Q–R) mixture (21.60–80.35%). QR-NIOs showed the lowest cumulative release throughout the experimental period, rising from only 3.38% to 51.63% over the same period, without reaching a plateau. This attenuated initial release and progressive diffusion indicate sustained polyphenol retention by the niosomal system. Nevertheless, dialysis measures membrane passage rather than gastrointestinal bioaccessibility. Similar retardation of quercetin release after niosomal encapsulation has been reported, supporting the carrier-mediated modulation observed here [22]. The Korsmeyer–Peppas model adequately fitted all profiles (R^2^ = 0.979–0.991; Figure 7B). QR-NIOs exhibited the lowest kinetic constant (k = 0.082) and exponent (*n* = 0.318), supporting the slowest apparent release among formulations. The time points, release intervals, and fitted parameters for each formulation are reported in Table S2. Free QUE and RES showed higher k values (0.241 and 0.248), whereas Q–R was intermediate (k = 0.194), directly consistent with the cumulative release profiles observed experimentally.

These kinetic parameters reinforce the experimental release profiles: the lower apparent k of QR-NIOs corresponded with their attenuated initial release and 51.63% diffusion after 24 h. For spherical systems, n ≈ 0.43 represents the Fickian benchmark; therefore, *n* = 0.318 is compatible with quasi-Fickian transport [22,31]. However, mechanistic interpretation requires fitting only the initial 60% of release. Additionally, dialysis-membrane resistance means that the model supports sustained apparent release but cannot demonstrate that niosomal-bilayer diffusion was the sole transport-controlling process. Lu et al. [22] likewise observed that free quercetin was released more rapidly than quercetin-loaded niosomes, supporting the capacity of niosomal formulations to delay apparent release. This qualitative pattern agrees with our 24 h dialysis results, in which cumulative passage was 97.52% for free quercetin, 80.35% for the unencapsulated 50:50 mixture, and 51.63% for QR-NIOs. However, Lu et al. evaluated a single-compound formulation intended for skin delivery in a different release medium; therefore, their release rates cannot be directly compared with those of our dual-loaded formulation, nor do the two studies establish the same transport mechanism.

#### 2.2.4. Incorporation into Functional Hummus

##### Microbiological Quality and Shelf-Life Stability

QR-NIO-enriched hummus maintained microbiological quality throughout 28 days of refrigerated storage at 4 ± 1 °C. Aerobic mesophilic bacteria, yeasts and molds, total and fecal coliforms, and Enterobacteriaceae remained below the analytical detection limits (<10 CFU/g [Colony-forming units per gram]). *Salmonella* spp. and *Listeria monocytogenes* were absent in 25 g, while *Staphylococcus aureus*, *Bacillus cereus*, and *Clostridium perfringens* were not detected. No detectable increase in microbial counts or evidence of contamination was observed at any of the sampling times during the evaluated storage period.

Microbial counts remaining below the detection limits and the absence of pathogens support the microbiological quality of the product throughout the 28-day period. Karam et al. evaluated hummus using microbial counts and sensory acceptability, confirming that shelf-life stability should integrate microbiological and sensory criteria rather than microbial safety alone [32].

##### Phenolic and Flavonoid Bioaccessibility During Simulated Gastrointestinal Digestion

**Figure 8:**
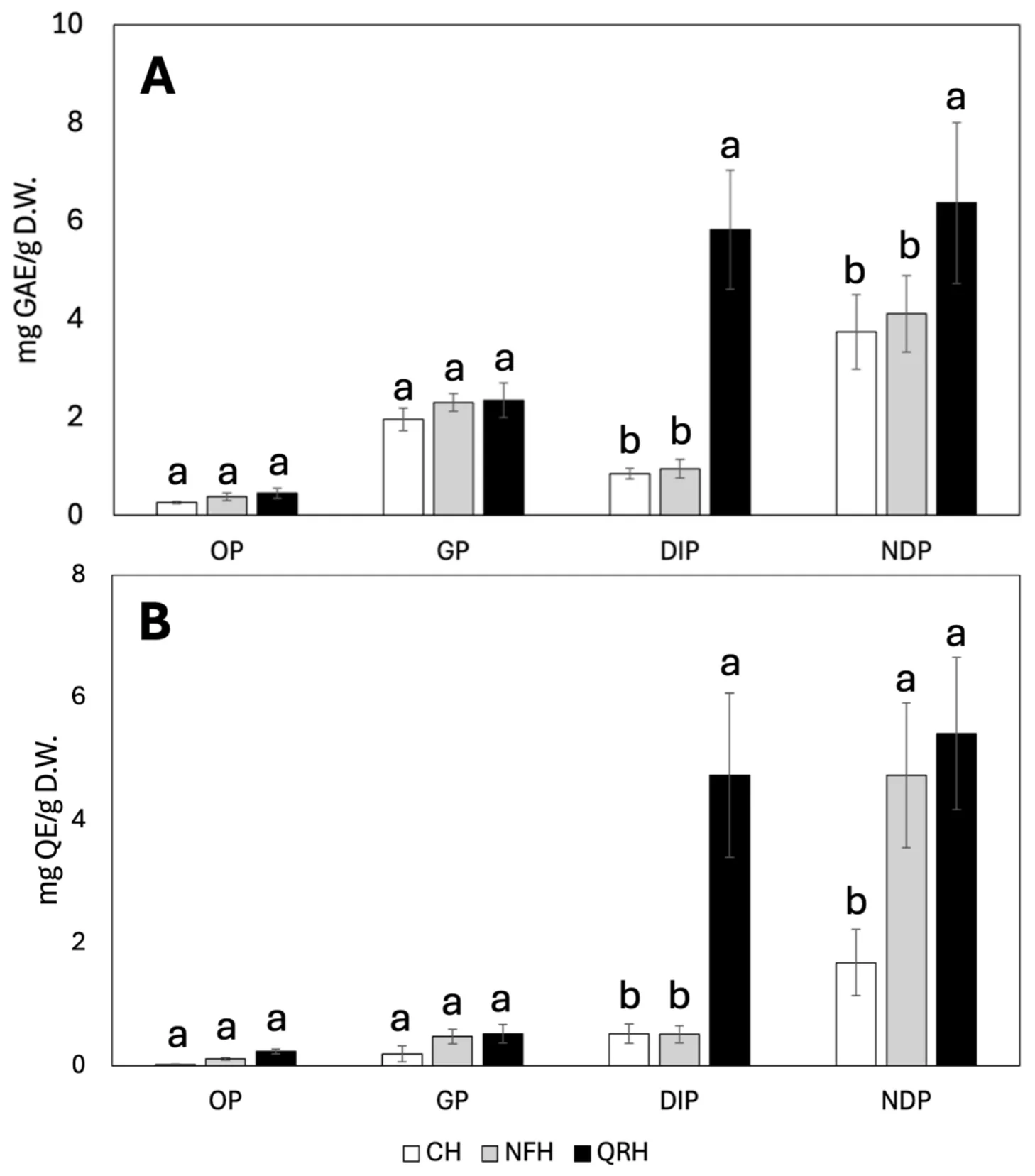
Total phenolic and flavonoid contents of hummus formulations during simulated gastrointestinal digestion. (**A**) Total phenolic content, expressed as milligrams of gallic acid equivalents per gram of dry weight (mg GAE/g D.W.). (**B**) Total flavonoid content, expressed as milligrams of quercetin equivalents per gram of dry weight (mg QE/g D.W.). CH, commercial hummus; NFH, non-functionalized hummus; QRH, QR-NIO-enriched hummus. OP, oral phase; GP, gastric phase; DIP, dialyzable intestinal fraction; NDP, non-dialyzable intestinal fraction. Values are presented as mean ± standard deviation (*n* = 3). Within each panel and each digestion phase or intestinal fraction, bars sharing at least one lowercase letter do not differ significantly, whereas bars with no letters in common differ significantly (*p* < 0.05). Letters are not compared across phases, fractions, or panels.

Total phenolic content was comparable among commercial hummus (CH), non-functionalized hummus (NFH), and QR-NIO-enriched functional hummus (QRH) during oral and gastric digestion (Figure 8). In the dialyzable intestinal fraction (DIP), QR-NIO-enriched functional hummus (QRH) reached approximately 5.8 mg GAE/g D.W., significantly exceeding both controls, and remained clearly highest in the non-dialyzable intestinal fraction (NDP) at approximately 6.3 mg GAE/g D.W. Total flavonoid content showed the same phase-dependent response. Oral phase (OP) and gastric phase (GP) values did not differ significantly among formulations, whereas QRH increased to approximately 4.7 mg QE/g D.W. in DIP, surpassing CH and NFH. In NDP, QRH and NFH retained similarly high contents and exceeded CH, indicating that enrichment enhanced the potentially bioaccessible fraction while leaving a substantial non-dialyzable flavonoid reservoir.

The intestinal increase agrees with Perez-Perez et al. [33], who found that digestive hydrolysis enhanced the bioaccessibility of free and conjugated phenolics in cooked chickpeas. Similarly, Yang et al. [18] reported that co-encapsulating quercetin and resveratrol delayed their gastrointestinal release while increasing in vitro bioaccessibility. Thus, the elevated DIP values in QRH may reflect matrix disruption combined with nanocarrier-mediated solubilization. Nevertheless, dialysis represents potential bioaccessibility rather than epithelial uptake or systemic bioavailability.

##### Antioxidant Capacity During Simulated Gastrointestinal Digestion

Antioxidant capacity increased markedly during intestinal digestion, although the strongest formulation depended on the assay (Table 8). NFH exhibited the highest DPPH radical inhibition (98.30%), whereas NFH and QRH showed statistically comparable ABTS radical inhibition values (91.84% and 93.64%, respectively). QRH nevertheless achieved the highest AAPH inhibition (89.63%), ferric reducing antioxidant power (FRAP) value (4233.41 µmol TE/g D.W.), and *N*,*N*-dimethyl-p-phenylenediamine (DMPD) response (2742.86 µmol TE/g D.W.). QRH also maintained substantial activity in DIP, reaching 82.22% DPPH, 85.64% ABTS, 76.26% AAPH, 2304.76 µmol TE/g D.W. FRAP, and 2576.19 µmol TE/g D.W. DMPD. CH remained consistently lower, particularly for AAPH, FRAP, and DMPD intestinal responses. Thus, QRH’s main advantage was not universal dominance in every assay, but the broadest antioxidant performance across complementary radical-scavenging and reducing-capacity mechanisms after intestinal digestion.

**Figure 9:**
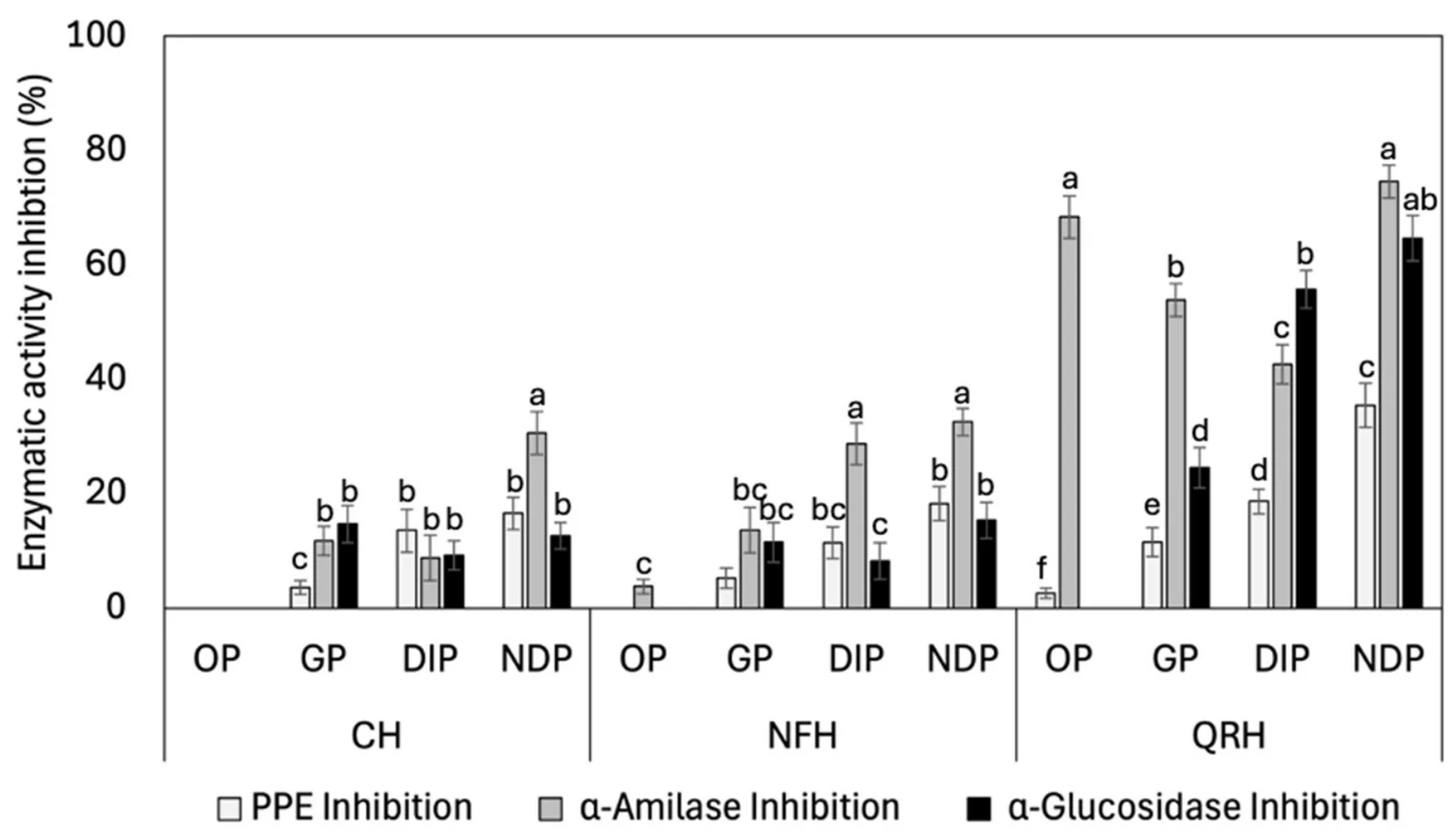
Inhibition of porcine pancreatic elastase (PPE), α-amylase, and α-glucosidase by CH, NFH, and QR-NIO-enriched hummus (QRH) during simulated gastrointestinal digestion. OP, oral phase; GP, gastric phase; DIP, dialyzable intestinal fraction; NDP, non-dialyzable intestinal fraction. Values are expressed as mean inhibition (%) ± standard deviation. Different lowercase letters indicate significant differences among digestion stages within each formulation and assay (*p* < 0.05); shared letters indicate no significant differences (*p* ≥ 0.05).

These patterns align with the phenolic and flavonoid results in Figure 9: QRH contained the largest dialyzable phenolic and flavonoid pools and showed strong DIP antioxidant activity. They also complement the earlier PBS release experiment, where QR-NIOs released only 51.63% after 24 h, indicating delayed diffusion. Taken together, the data suggest that niosomal retention limited immediate availability, whereas intestinal pH, pancreatin, bile salts, and matrix disruption promoted later liberation or solubilization of quercetin and resveratrol. The high antioxidant activity of the non-dialyzable intestinal fraction of QR-NIO-enriched hummus (QRH-NDP) suggests that compounds with antioxidant activity remained in the non-dialyzable fraction and could potentially reach the colon. Similar increases in digestive bioaccessibility after quercetin–resveratrol co-encapsulation have been reported, although compound-specific chromatography is required to attribute these responses directly to either polyphenol [18].

Physiologically, the dialyzable antioxidants may increase luminal exposure and potential epithelial availability, while the non-dialyzable reservoir could support later colonic metabolism [34]. Nanoparticle-delivered resveratrol has shown improved intestinal transport, plasma exposure, and anti-inflammatory activity [33]; however, the present chemical assays cannot establish absorption, metabolism, safety, or therapeutic efficacy in humans.

##### Porcine Pancreatic Elastase, α-Amylase, and α-Glucosidase Inhibition During Simulated Gastrointestinal Digestion

Measuring PPE, α-amylase, and α-glucosidase inhibition provides complementary evidence of the functional potential retained after digestion. PPE inhibition indicates the capacity to modulate proteolytic activity associated with inflammatory processes, whereas inhibition of α-amylase and α-glucosidase may delay sequential carbohydrate hydrolysis and glucose release, supporting the evaluation of anti-inflammatory and glycemic-modulating functional food applications. The corresponding results are presented in Figure 9.

PPE inhibition increased during digestion in all hummus formulations. CH rose from undetectable activity at OP to approximately 16% at NDP, while NFH increased from negligible activity to about 18%. QRH showed the strongest numerical response, progressing from nearly 2% at OP to 11% at GP, 18% at DIP, and 35% at NDP, its highest value observed in the assay. α-Amylase inhibition showed clear digestion- and formulation-dependent changes. CH increased from undetectable at OP to approximately 30% at NDP, whereas NFH rose from about 4% to 32%. QRH exhibited the highest numerical activities, with 68% at OP, 53% at GP, 42% at DIP, and 74% at NDP. OP and NDP shared the highest statistical group within QRH for this assay. α-Glucosidase inhibition was undetectable during OP but emerged after gastric digestion. CH remained near 9–14% from GP through NDP, while NFH reached its maximum of approximately 15% at NDP. QRH increased markedly from 24% at GP to 55% at DIP and 64% at NDP; the shared letters indicated that its DIP and NDP responses were not significantly different within QRH.

Across all three assays, QRH exhibited the strongest inhibitory profile, particularly in NDP, supporting digestion-dependent retention of multifunctional quercetin–resveratrol activity with potential intestinal and colonic relevance for functional food applications.

##### Sensory Acceptance of Functional Hummus

Sensory evaluation showed high acceptance of CH, NFH, and QRH, with QRH receiving the strongest visual ratings (Table 9). QRH achieved significantly higher appearance (8.35) and color (7.76) scores than CH (7.24 and 6.43) and NFH (7.41 and 6.64), respectively. Aroma and flavor remained highly rated and statistically comparable across formulations; QRH recorded the highest numerical values for both attributes (8.67 and 8.47). These results demonstrate that QR-NIO enrichment improved visual appeal without compromising the characteristic aroma or flavor of hummus. Although variability was greater for QRH aroma, its overall sensory profile indicates successful integration of the nanovesicles into the food matrix.

QRH also achieved the highest bitterness-acceptance (8.74) and mouthfeel (7.56) scores, remaining comparable to CH but exceeding NFH. Texture did not differ among formulations, while spread ability favored CH (7.66) over QRH (7.37) and NFH (6.91). Overall acceptability was numerically highest for CH (8.32), followed closely by QRH (8.22) and NFH (7.83), without a supported significant difference. Therefore, QRH was the best-performing functional formulation, combining enhanced appearance, color, bitterness acceptance, and mouthfeel with overall sensory liking comparable to the commercial hummus.

## 3. Discussion

Antioxidant activity varied with the quercetin (Q) molar ratio and the analytical method in both the free mixtures and their corresponding niosomal formulations. The 100:0 ratio produced the highest DPPH response in each group, although free quercetin showed significantly greater activity than its niosomal formulation. In contrast, the 50:50 and 75:25 ratios produced the highest ABTS responses, without significant differences between these ratios or between their free and niosomal counterparts. This finding indicates that ABTS radical-scavenging activity was largely preserved following encapsulation; it does not demonstrate an overall enhancement of antioxidant activity by niosomes. Differences between the DPPH and ABTS patterns are consistent with their distinct reaction mechanisms and assay conditions and with previous evidence that quercetin–resveratrol interactions vary across redox assays [35,36]. Quercetin’s catechol B ring and conjugated C ring facilitate electron or hydrogen donation and radical delocalization, whereas resveratrol forms a differently stabilized stilbene-derived radical. Collectively, these findings support assay-dependent antioxidant behavior but do not establish universal quercetin–resveratrol synergy or biological protection in vivo.

The chemical antioxidant findings were partially reflected in the erythrocyte membrane model. The 50:50 formulation afforded the highest inhibition of AAPH-induced hemolysis in A RhD+ erythrocytes and produced the strongest response in B RhD+ cells, whereas other ratios performed better in AB and O phenotypes. Microscopic observations were consistent with these measurements: treated A RhD+ erythrocytes largely retained a discocyte-like morphology, whereas O RhD− cells showed residual deformation. Quercetin has previously been reported to increase erythrocyte resistance to AAPH-mediated damage [37], and ABO/RhD-dependent responses have also been described [24]. Nevertheless, these results were obtained in an ex vivo membrane model and should not be interpreted as evidence of systemic protection.

The 50:50 formulation showed the highest mean PPE inhibition and retained the greatest relative inhibitory activity over the 60 min assay. It was also among the most active formulations against both α-amylase and α-glucosidase. Previous studies have described interactions between polyphenols and PPE [26] and reported assay-dependent modes of carbohydrate-hydrolyzing enzyme inhibition by quercetin, resveratrol, and other polyphenols [26,38,39,40,41]. These findings provide context for the observed activities, but the present assays did not determine binding sites or modes of inhibition. Accordingly, the results demonstrate inhibitory activity against multiple enzymes under the tested in vitro conditions.

The multifunctional profile identified in this study was not produced by a single universal interaction but by endpoint-specific relationships between quercetin and resveratrol. The 50:50 ratio was essentially additive in the DPPH assay but synergistic in the ABTS assay, whereas synergistic erythroprotection was observed only in A RhD+ and B RhD+ erythrocytes. Such variations are consistent with evidence that phenolic mixtures may shift among synergistic, additive, and antagonistic interactions depending on radical chemistry, concentration, and cellular redox conditions [35,36]. Electron or hydrogen transfer, regeneration of oxidized intermediates, and differential membrane partitioning may contribute to these responses, although these mechanisms were not directly investigated. Therefore, the principal contribution of this optimization was the identification of an equimolar formulation with the broadest favorable response across chemically and biologically distinct experimental systems.

The enzyme interaction analysis further demonstrated this context dependence. After correction of the expected additive values, all mixed ratios showed synergistic PPE inhibition according to the predefined interaction index thresholds. The 50:50 formulation produced the highest absolute PPE inhibition (57.86%; II = 1.957), whereas the 25:75 formulation exhibited the greatest proportional enhancement relative to its expected additive response (53.56% versus 23.99%; II = 2.233). In the α-amylase assay, the 50:50 formulation also showed the highest experimental inhibition (93.23%), exceeding its expected additive value and meeting the predefined criterion for synergy (II = 1.061). For α-glucosidase, the 50:50 formulation similarly showed modest synergy. Polyphenol–enzyme interactions involving hydrogen bonding and hydrophobic contacts are plausible [26,42]; however, an interaction index above unity does not identify the binding site, inhibition mechanism, or statistical significance of the interaction.

The broad functional profile of the 50:50 ratio becomes technologically relevant only if both polyphenols are effectively retained within the niosomal preparation. The rounded-to-ellipsoidal morphology, overall encapsulation efficiency of 90.63 ± 3.63%, hydrodynamic diameter of approximately 149 nm, low polydispersity index, and ζ-potential of approximately −34 mV collectively support nanoscale vesicle formation and initial colloidal stability. Span 60 likely provided a relatively ordered hydrophobic bilayer domain, whereas Tween 80 and cholesterol may have modulated interfacial hydration, membrane packing, and permeability; however, cholesterol–surfactant effects are strongly dependent on formulation composition [43,44]. Quercetin and resveratrol may partition at different depths within the bilayer through hydrogen bonding, hydrophobic association, van der Waals forces, or π–π interactions, but their respective locations were not determined. Furthermore, the individual encapsulation values obtained from separately prepared single-compound niosomes demonstrate the compatibility of each compound with the carrier but do not provide compound-specific encapsulation efficiencies for the jointly formulated 50:50 QR-NIOs. SEM also examined dehydrated particles and showed a larger and more heterogeneous population than that measured by DLS. Therefore, the morphology and overall encapsulation results support vesicle formation and polyphenol retention but do not demonstrate simultaneous localization of both compounds in every individual niosome.

The physicochemical profile provides a plausible explanation for the attenuated initial release and the 51.63% cumulative passage measured after 24 h, because association with the niosomal bilayer may delay partitioning into the aqueous release medium. The lower Korsmeyer–Peppas rate constant (k = 0.082) and the strong model fit (R^2^ = 0.991) quantitatively describe the observed sustained-release profile, consistent with previous studies of quercetin-loaded niosomes [20,39]. However, the release exponent (*n* = 0.318) was below the spherical Fickian benchmark of 0.43 [31]. In addition, the Korsmeyer–Peppas model was originally developed for matrix systems, and resistance imposed by the dialysis membrane may have contributed to the measured profile. Consequently, the present experiment cannot independently establish bilayer-controlled diffusion as the release mechanism.

Incorporation of QR-NIOs into hummus was subsequently evaluated to determine whether the formulation could be introduced into a complex food matrix while maintaining technological feasibility. Microbial counts below the detection limit and the absence of the evaluated pathogens over 28 days at 4 °C support adequate processing hygiene and refrigerated stability. Nevertheless, these findings cannot be attributed specifically to an antimicrobial effect of QR-NIOs because an inoculated challenge test and a matched longitudinal control capable of establishing causality were not included. This distinction is relevant because hummus may support spoilage or pathogen persistence, while its microbial stability depends on several factors, including acidity, packaging, refrigeration, and hygienic processing [32].

The sensory findings provide evidence of technological compatibility. The mixed-effects analysis, which accounted for repeated evaluations by the same 30 panelists, showed that QRH received higher scores than both CH and NFH for appearance and color. Aroma, mouthfeel, spreadability, and overall acceptability did not differ significantly between QRH and the commercial hummus, whereas QRH performed more favorably than NFH for these attributes. Flavor and texture showed smaller formulation-dependent differences, with QRH generally remaining comparable to CH. Bitterness acceptance was also comparable between QRH and CH and was higher than that of NFH. These results indicate that incorporation of QR-NIOs did not produce an evident sensory penalty relative to the commercial product. However, the suggestion that encapsulation or the hummus matrix masked polyphenol bitterness remains hypothetical because perceived bitterness intensity and sensory masking were not measured directly.

The digestion results showed similar assay-estimated phenolic and flavonoid contents during the oral and gastric phases, followed by higher values in the dialyzable intestinal fraction of QRH. This pattern is consistent with the formation or release of additional Folin-reactive and flavonoid-reactive compounds during intestinal digestion. Pancreatin-mediated matrix hydrolysis, intestinal pH, and bile salts may disrupt or reorganize niosomes and facilitate the transfer of hydrophobic compounds into mixed colloidal structures. Similar co-encapsulation systems have been reported to improve the apparent bioaccessibility of quercetin and resveratrol while delaying their gastrointestinal release [18,45]. Nevertheless, the colorimetric assays used here cannot distinguish intact quercetin and resveratrol from their transformation products or from other compounds released from the hummus matrix. Therefore, the observed increases should not be interpreted as compound-specific recovery or direct evidence of intact-polyphenol bioaccessibility.

The intestinal fractions retained antioxidant and enzyme-inhibitory activity, linking the physicochemical changes occurring during digestion with preserved in vitro functionality. QRH-NDP showed high AAPH, FRAP, and DMPD responses, whereas NFH produced the highest DPPH response, confirming that relative performance depended on the analytical method. Phenolic compounds and digestion products may donate electrons or hydrogen atoms, stabilize radical intermediates, and interrupt radical propagation, while chickpea-derived phenolics, peptides, and other matrix components may also contribute to the measured responses. QRH-NDP also inhibited PPE, α-amylase, and α-glucosidase. These activities may involve hydrogen bonding and hydrophobic interactions with the enzymes [39], but neither the responsible compounds nor the mechanisms of inhibition were determined. The persistence of bioactive chickpea peptides and isoflavones following digestion [46] further supports the likely contribution of interactions between the food matrix and the nanocarrier system.

Physiologically, the dialyzable intestinal fraction may approximate the fraction potentially available for contact with the small-intestinal epithelium, whereas the non-dialyzable fraction may contain compounds capable of reaching the colonic microbiota. However, dialysis does not reproduce epithelial transport, metabolism, tissue distribution, or systemic bioavailability. Absorbed quercetin and resveratrol would also undergo extensive glucuronidation, sulfation, and methylation [47], meaning that circulating metabolites may differ substantially from their parent compounds. Consequently, the antioxidant and enzyme-inhibition assays indicate potential luminal functionality but do not establish anti-inflammatory effects, glycemic control, obesity prevention, cardiovascular protection, or clinical efficacy.

Several analytical limitations should be considered. UV–Vis spectroscopy and total phenolic and flavonoid assays provided useful comparative measurements but lacked the molecular specificity required to distinguish quercetin, resveratrol, their degradation products, and compounds originating from the hummus matrix. Similarly, the reported overall encapsulation efficiency does not replace separate quantification of quercetin and resveratrol in the jointly loaded formulation. The simulated digestion model also did not include epithelial absorption or metabolism. High-performance liquid chromatography (HPLC) or liquid chromatography–mass spectrometry (LC–MS) analysis would therefore be necessary to confirm compound-specific encapsulation, release, transformation, and recovery following gastrointestinal digestion. These analyses could not be performed in the present study because the required chromatographic instrumentation was unavailable.

In food applications, QRH represents a sensory-compatible approach for incorporating poorly water-soluble polyphenols into a plant-based dip. Its digestion-dependent chemical and functional responses support further investigation as a functional food formulation, provided that dose, storage stability, compound-specific bioaccessibility, and safety are established. QR-NIOs may also provide an experimental oral co-delivery platform for investigating ratio-dependent antioxidant and enzyme-modulating effects [48]. Nevertheless, the present findings support additional preclinical and clinical investigation rather than therapeutic recommendations. Future studies should quantify quercetin, resveratrol, and their metabolites using HPLC or LC–MS, evaluate incorporation into mixed micelles and epithelial transport using appropriate cellular models, perform microbiological challenge testing, investigate toxicity and dose–response relationships, and assess postprandial glycemia, inflammatory biomarkers, pharmacokinetics, and tolerability in controlled human studies.

## 4. Materials and Methods

### 4.1. Materials and Reagents

Quercetin, resveratrol, Span 60, Tween 80, and cholesterol were used to prepare the niosomal formulations. Reagents for the antioxidant assays included Trolox, DPPH, ABTS, 2,4,6-tripyridyl-s-triazine (TPTZ), *N*,*N*-dimethyl-p-phenylenediamine (DMPD), and ferric chloride. Porcine pancreatic elastase, α-amylase, α-glucosidase, and their corresponding assay substrates were used for the enzyme-inhibition experiments. The listed chemicals, reagents, and enzymes were purchased from Sigma-Aldrich. Dialysis membranes were used for the release and intestinal-fractionation procedures. Hummus was prepared from cooked chickpeas, tahini, extra virgin olive oil, water, lemon juice, sodium chloride, and paprika.

### 4.2. Ethical Handling of Human Erythrocyte Membrane-Based Assays

All procedures involving human erythrocytes were conducted under institutional ethical supervision and in accordance with the approved study protocol. Erythrocyte samples were supplied by the University of Guadalajara Clinical Analysis Laboratory. Blood collection and sample handling followed the laboratory’s established procedures. The laboratory operates under ISO/IEC 17025 (NMX-EC-17025) and ISO 15189 accreditation and applies the quality-management procedures described in the cited institutional documentation [49,50,51,52,53].

Peripheral venous blood was collected from healthy adults aged 20–40 years whose erythrocyte counts were within the physiological range (4.7–6.1 × 10^6^ cells/µL). Participants were informed about the study and provided written informed consent before sample collection. Blood was collected in sterile vacuum tubes containing ethylenediaminetetraacetic acid (EDTA), and the isolated erythrocytes were used to evaluate the protection mediated by quercetin, resveratrol, and QR-NIO against AAPH-induced oxidative membrane damage. The study followed the ethical principles of the Declaration of Helsinki and was reviewed and approved by the Research Ethics Committee of the University Center of the Valleys, University of Guadalajara (protocol CI 2023-47; registration CEI/104/2025; certificate CUVALLES/CI-CEI/97-1/2025).

### 4.3. Preparation of Free Quercetin–Resveratrol Mixtures at Different Q Ratios

Individual stock solutions of quercetin and resveratrol were prepared in dimethyl sulfoxide (DMSO) because of their limited aqueous solubility. Free, unencapsulated mixtures were prepared at five quercetin-to-resveratrol (Q) molar ratios and a total polyphenol concentration of 4 mM: 100:0 (4:0 mM), 75:25 (3:1 mM), 50:50 (2:2 mM), 25:75 (1:3 mM), and 0:100 (0:4 mM). The mixtures were diluted to the concentrations required for the respective biological assays (12.5, 25, 50, 100, and 200 µg/mL, as applicable). The final DMSO concentration in the assay samples was 1% (*v*/*v*), and vehicle controls contained the same DMSO concentration. These free mixtures were evaluated alongside niosomal formulations prepared at the corresponding Q ratios.

### 4.4. Antioxidant Activity Assays

Antioxidant activity was assessed using the DPPH and ABTS radical-scavenging assays, the ferric reducing antioxidant power (FRAP) assay, and the *N*,*N*-dimethyl-p-phenylenediamine (DMPD) radical-cation decolorization assay. For DPPH [54], 20 µL of sample was mixed with 200 µL of DPPH solution adjusted to an absorbance of 0.70 ± 0.02 at 515 nm; after 30 min in the dark, absorbance was measured at 515 nm. For ABTS [55], 20 µL of sample was combined with 270 µL of ABTS•^+^ solution adjusted to an absorbance of 0.70 ± 0.02 at 734 nm; the mixture was kept in the dark for 30 min before measurement at 734 nm.

For FRAP [56], a working reagent was prepared from sodium acetate buffer, 2,4,6-tripyridyl-s-triazine (TPTZ) in hydrochloric acid, and ferric chloride; the increase in absorbance associated with formation of the ferrous–TPTZ complex was measured at 638 nm after 30 min. For DMPD [57], 1 mL of 100 mM DMPD solution was added to 100 mL of 0.1 M acetate buffer (pH 5.25), followed by 0.2 mL of 0.05 M FeCl_3_ to generate DMPD•^+^. The fresh reagent was adjusted to an initial absorbance of approximately 0.90 ± 0.10 at 505 nm. Each well received 180 µL of reagent and 20 µL of sample or Trolox standard; after 10 min at room temperature in the dark, the decrease in absorbance was measured at 505 nm. Measurements were performed in triplicate. For the Q screening, DPPH and ABTS results were calculated from Trolox calibration curves and expressed as µmol Trolox equivalents per gram of dry weight (µmol TE/g D.W.). For the digestion fractions, DPPH and ABTS results were expressed as inhibition (%), whereas FRAP and DMPD results were expressed as µmol TE/g D.W.

### 4.5. Selection of the Q:R Ratio

#### 4.5.1. Selection of the Quercetin:resveratrol Niosome Ratio

Niosomal formulations were prepared at each of the five Q:R molar ratios described in Section 4.3 using a modified ultrasound-assisted thin-film hydration method [58,59]. For each 20 mL batch, Span 60 (400 mg; 2.0% *w*/*v*), Tween 80 (200 mg; 1.0% *w*/*v*), and cholesterol (20 mg; 0.1% *w*/*v*) were dissolved in food-grade ethanol. Quercetin and resveratrol were added from their DMSO stock solutions in amounts corresponding to a total nominal polyphenol concentration of 4 mM: 100:0 (4:0 mM), 75:25 (3:1 mM), 50:50 (2:2 mM), 25:75 (1:3 mM), or 0:100 (0:4 mM). The mixture was stirred until homogeneous and subjected to rotary evaporation at 45 °C and 100 rpm under reduced pressure (250–300 mbar) for approximately 10 min, or until a uniform thin film formed. The film was further dried under vacuum, hydrated with phosphate-buffered saline (PBS, pH 7.4), and adjusted to a final suspension volume of 20 mL.

The hydrated vesicles were processed using a probe-type ultrasonic processor (Benchmark Scientific, Inc., Sayreville, NJ, USA) equipped with a 10 mm-diameter horn. Sonication was performed at a nominal frequency of 25 kHz and an amplitude setting of 65%, with alternating 2 s-on and 2 s-off pulses for a programmed time of 15 min. The recorded probe temperature was approximately 23 °C. The five niosomal formulations were evaluated in the ratio-screening assays alongside their corresponding free mixtures. Based on the combined results of the ratio-screening assays, the 50:50 niosomal formulation was selected for further study. Its biological activities were reassessed, and it was characterized by scanning electron microscopy (SEM), average particle size, polydispersity index (PDI), ζ-potential, and encapsulation efficiency measurements. This selected formulation was subsequently used to enrich the hummus.

#### 4.5.2. Antioxidant Screening of Free Quercetin–Resveratrol Mixtures and Their Niosomal Formulations

The antioxidant interactions of dual-loaded quercetin–resveratrol niosomes (QR-NIOs) were investigated to identify the formulation exhibiting the greatest synergistic antioxidant effect and to determine the optimal quercetin–resveratrol ratio. Antioxidant activity was assessed using two complementary methods: DPPH and ABTS, which evaluate different mechanisms of antioxidant action, including free radical scavenging and reducing power. For the DPPH assay, 20 µL of each sample was mixed with 200 µL of DPPH solution adjusted to an absorbance of 0.70 ± 0.02 at 515 nm and incubated in darkness for 30 min. In the ABTS assay, 20 µL of sample was combined with 270 µL of ABTS•+ solution adjusted to an absorbance of 0.70 ± 0.02 at 734 nm and maintained in the dark for 30 min.

All measurements were performed in triplicate. Antioxidant activity was expressed as µmol Trolox equivalents per gram (µmol TE/g) for DPPH and ABTS assays. For the DPPH and ABTS assays, antioxidant activity was calculated using the following equation:

(1) $$\mathrm{Inhibition}\;(\%)=\frac{A_{\mathrm{control}}-A_{\mathrm{Sample}}}{A_{\mathrm{control}}}\times 100$$

where *A_control_* is the measured absorbance of the control reaction without the test sample and *A_sample_* is the measured absorbance of the reaction containing the test sample. Results were expressed as the percentage of elastase inhibition relative to the control.

#### 4.5.3. Inhibition of AAPH-Induced Oxidative Damage in Human Erythrocytes

The erythroprotective activity of different dual-loaded quercetin–resveratrol niosome (QR-NIO) formulations was evaluated using an AAPH-induced oxidative hemolysis assay to identify the formulation exhibiting the greatest membrane-protective and antioxidant effects. Quercetin–resveratrol mixtures (100:0, 75:25, 50:50, 25:75, and 0:100, *w*/*w*) were prepared at concentrations of 12.5, 25, 50, 100, and 200 μg/mL. Human erythrocytes obtained from healthy adult volunteers were washed three times with phosphate-buffered saline (PBS, pH 7.4) and adjusted to a final cell suspension of 2% (*v*/*v*).

The reaction mixture consisted of 100 μL of erythrocyte suspension, 100 μL of the corresponding quercetin–resveratrol combination, and 100 μL of freshly prepared AAPH solution (40 mM). Oxidative controls contained erythrocytes incubated with AAPH alone, whereas negative controls received PBS instead of AAPH. After incubation at 37 °C for 3 h under gentle agitation (45 rpm), samples were centrifuged (1500 rpm, 10 min), and hemolysis was quantified by measuring the absorbance of the supernatant at 540 nm. Erythroprotective activity was expressed as the percentage inhibition of AAPH-induced hemolysis relative to the oxidative control, and the combination showing the highest protective effect was selected for subsequent niosome formulation [60]. The inhibition of AAPH-induced oxidative damage in human erythrocytes was calculated using the following equation:

(2) $$\mathrm{Hemolysis}\mathrm{Inhibition}\left(\%\right)=\frac{\left(A_{\mathrm{Cpos}-}\left(A_{\mathrm{TS}}-A_{\mathrm{Cneg}}\right)\right)}{A_{\mathrm{Cpos}}}\times 100$$

where *A_Cpos_* is the absorbance of the positive control (erythrocytes exposed to AAPH without a test formulation), *A_TS_* is the absorbance of erythrocytes exposed to AAPH in the presence of the test formulation, and *A_Cneg_* is the absorbance of the negative control (erythrocytes not exposed to AAPH). Absorbance at 540 nm reflects hemoglobin released into the supernatant and was used as an indicator of hemolysis. Thus, lower hemoglobin release in the treated sample corresponds to greater inhibition of AAPH-induced hemolysis.

As a complementary study, erythrocyte morphology was microscopically evaluated to provide qualitative evidence of the protective effects of dual-loaded quercetin–resveratrol niosomes (QR-NIOs) against AAPH-induced oxidative damage through the preservation of erythrocyte membrane integrity. After incubation, erythrocytes were collected by centrifugation, and thin blood smears were prepared on clean glass slides, air-dried, and stained with Wright’s stain according to standard hematological procedures. The stained smears were examined using an Eclipse FN1 optical microscope (Nikon Instruments Inc., Tokyo, Japan) equipped with NIS-Elements imaging software (version 6.1) at 100× magnification under oil immersion. Representative micrographs were obtained for the positive control (AAPH-induced hemolysis), the negative control (untreated erythrocytes), and the QR-NIO formulation that demonstrated the greatest protective effect against AAPH-induced hemolysis among the evaluated quercetin–resveratrol combinations [22,24,60].

#### 4.5.4. Porcine Pancreatic Elastase (PPE) Inhibition Assay

The anti-inflammatory activity of dual-loaded quercetin–resveratrol niosomes (QR-NIOs) was evaluated by measuring their inhibitory effect on porcine pancreatic elastase (PPE, Type IV, Sigma-Aldrich, St. Louis, MO, USA), following the method described by Lee et al. [61] with minor modifications. Elastase activity was determined using N-succinyl-Ala-Ala-Ala-p-nitroanilide (1.015 mM) as substrate prepared in Tris-HCl buffer (0.1 M, pH 8.0), while the PPE enzyme was prepared at 1.376 U mL^−1^ in Tris-HCl buffer (0.2 M, pH 8.0). The reaction mixture consisted of 10 μL of the corresponding QR-NIO formulation, 40 μL of PPE solution, and 100 μL of substrate solution, giving a final reaction volume of 150 μL. Absorbance was monitored at 410 nm for 60 min at 28 °C using a microplate reader.

All determinations were performed in triplicate, and elastase inhibition was expressed as the percentage inhibition relative to the enzyme control. The QR-NIO formulation exhibiting the highest inhibitory activity was selected for subsequent physicochemical characterization and biological evaluation. Elastase inhibition was calculated according to the following equation:

(3) $$\mathrm{Inhibition}\left(\%\right)=\left(1-\frac{A}{B}\right)\times 100$$

where A is the absorbance of the control and B is the absorbance in the presence of the extract. Results were expressed as percentage inhibition of elastase activity.

#### 4.5.5. α-Amylase and α-Glucosidase Inhibition

The α-amylase and α-glucosidase inhibitory activities of dual-loaded quercetin–resveratrol niosomes (QR-NIOs) were assessed by evaluating their inhibitory activity against the carbohydrate-digesting enzymes α-amylase [62,63] and α-glucosidase [63,64]. For the α-amylase assay, 50 μL of each QR-NIO formulation was incubated with 50 μL of porcine pancreatic α-amylase (1 U/mL) prepared in 20 mM sodium phosphate buffer (pH 6.9) containing 6 mM NaCl at 37 °C for 10 min. Subsequently, 50 μL of 1% (*w*/*v*) soluble starch was added, and the reaction proceeded for 15 min before termination with 100 μL of 3,5-dinitrosalicylic acid (DNS) reagent. After heating at 95 °C for 5 min and cooling to room temperature, the mixtures were diluted with distilled water, and absorbance was measured at 540 nm. Acarbose and buffer served as positive and negative controls, respectively. The percentage of α-amylase inhibition was calculated using the following equation:

(4) $$\mathrm{Inhibition}\left(\%\right)=\left(1-\frac{A_{\mathrm{Sample}}}{A_{\mathrm{Control}}}\times 100\right)$$

The inhibitory effect of dual-loaded quercetin–resveratrol niosomes (QR-NIOs) against α-glucosidase was determined using p-nitrophenyl-α-D-glucopyranoside (pNPG) as the substrate. Briefly, 50 μL of each formulation was incubated with 50 μL of α-glucosidase solution (1 U/mL) prepared in 100 mM phosphate buffer (pH 6.8) at 37 °C for 10 min. The reaction was initiated by adding 50 μL of 5 mM pNPG and terminated after 20 min with 100 μL of 0.2 M sodium carbonate. Absorbance was recorded at 405 nm. Acarbose and buffer were used as positive and negative controls, respectively, and enzyme inhibition was expressed as a percentage using the following equation:

(5) $$\mathrm{Inhibition}\left(\%\right)=\left(1-\frac{A_{\mathrm{Sample}}}{A_{\mathrm{Control}}}\times 100\right)$$

#### 4.5.6. Interaction Index and Classification

The synergistic interaction between quercetin and resveratrol was determined by comparing the experimentally observed inhibitory activity of each combination with the theoretical additive effect calculated from the activities of the individual compounds at the corresponding concentrations [65,66]. The expected inhibitory activity (Eexpected) was calculated as:

(6) $$E_{\mathrm{expected}\;=\;\left(F_{q}\;-\;E_{q}\right)\;+\;\left(F_{r}\;-\;E_{r}\right)}$$

where FQ and FR represent the weight fractions of quercetin and resveratrol in each formulation, respectively, while EQ and ER correspond to the inhibitory activities of quercetin and resveratrol tested individually at the same total concentration. The interaction index (II) was subsequently calculated according to:

(7) $$\mathrm{II}=\frac{E_{\mathrm{experimental}}}{E_{\mathrm{expected}}}$$

Interaction index values greater than 1.0 were interpreted as synergistic effects, values close to 1.0 indicated additive interactions, and values lower than 1.0 suggested antagonistic behavior.

### 4.6. Characterization of the Selected 50:50 QR-NIO Formulation

#### 4.6.1. Scanning Electron Microscopy (SEM)

The morphology and surface characteristics of the selected dual-loaded quercetin–resveratrol niosomes (QR-NIOs) were examined by scanning electron microscopy (SEM), with adaptations from previously reported nanovesicle characterization procedures [67,68]. Before analysis, the niosomal dispersion was freeze-dried, and the resulting powder was mounted on aluminum stubs using conductive carbon tape. Samples were sputter-coated with a thin gold layer to minimize surface charging and improve image contrast. Micrographs were acquired using a JSM-6610LV scanning electron microscope (JEOL Ltd., Tokyo, Japan) under high-vacuum conditions at an accelerating voltage of 20 kV and working distances of 9–10 mm. Representative images were recorded at ×70,000 and ×80,000 magnification to assess apparent particle shape, surface topography, dry-state dimensions, morphological heterogeneity, and aggregation. SEM observations were interpreted together with dynamic light-scattering measurements because freeze-drying, vacuum exposure, and conductive coating may alter vesicle dimensions and morphology.

#### 4.6.2. Assessment of Average Particle Size, Polydispersity Index and ζ-Potential Stability and Encapsulation Efficiency

The physicochemical properties of the selected 50:50 dual-loaded quercetin–resveratrol niosomes (QR-NIOs) were evaluated by measuring their average particle size and polydispersity index (PDI) by dynamic light scattering (DLS) and their ζ-potential by electrophoretic light scattering. Briefly, 1.0 mL of the niosomal suspension was diluted in 100 mL of phosphate-buffered saline (PBS, pH 7.4) and transferred to disposable cuvettes for analysis [69]. Measurements were performed at room temperature using a Nano-ZS90 analyzer (Malvern Instruments Ltd., Malvern, UK).

The encapsulation efficiency of quercetin and trans-resveratrol was determined by size-exclusion chromatography followed by multicomponent UV–Vis spectrophotometry. A 2.5 mL aliquot of the QR-NIO dispersion was applied to a PD-10 column packed with Sephadex G-50 and equilibrated with phosphate-buffered saline (PBS; 10 mmol/L, pH 7.4). Sixteen 0.5 mL fractions were collected. Vesicle-containing fractions were distinguished from those containing free compounds by light scattering at 600 nm and particle-size analysis; their elution windows were established using empty niosomes and free-polyphenol controls. Vesicle-associated compounds were extracted with methanol. Quercetin and resveratrol were then quantified using absorbance measurements at 370 and 306 nm, respectively, and simultaneous equations derived from separate calibration curves for both compounds. The encapsulation efficiency of each compound was calculated from its amount in the vesicle-containing fractions relative to its total amount in an unpurified aliquot, with mass recovery assessed across the collected fractions. Analyses were performed in triplicate under reduced lighting to limit resveratrol photoisomerization. Overall apparent encapsulation efficiency was calculated on a mass basis as:

(8) $${\mathrm{EE}}_{\mathrm{Apparent}\mathrm{global}\mathrm{encapsulation}\mathrm{efficiency}}(\%)=\frac{m_{{\mathrm{QUE}}_{\mathrm{enc}}}{\;+\;m}_{{\mathrm{RES}}_{\mathrm{enc}}}}{m_{{\mathrm{QUE}}_{\mathrm{total}}}{\;+\;m}_{{\mathrm{RES}}_{\mathrm{total}}}}\times 100$$

where the apparent global encapsulation efficiency (%) represents the apparent percentage of the total polyphenol mass associated with the niosomal fraction; *m_QUEenc_* is the mass of encapsulated quercetin recovered from the vesicle-associated fractions; *m_RESenc_* is the mass of encapsulated resveratrol recovered from the vesicle-associated fractions; *m_QUEtotal_* is the total mass of quercetin initially present in the unpurified niosomal formulation; *m_REStotal_* is the total mass of resveratrol initially present in the unpurified niosomal formulation; and 100 is the conversion factor used to express the result as a percentage.

The apparent global encapsulation efficiency represents the combined mass fraction of quercetin and resveratrol associated with the niosomal population. This ensemble-level parameter represents the combined fraction of polyphenols associated with the niosomal population but does not demonstrate that both compounds are simultaneously encapsulated within each individual vesicle. All analyses were conducted in triplicate, and results were expressed as the mean ± standard deviation.

#### 4.6.3. In Vitro Release Study

The release profile of the optimal synergistic dual-loaded quercetin–resveratrol niosomes (QR-NIOs) was evaluated using a dialysis membrane diffusion method [70]. Briefly, 10 mL of the niosomal suspension was placed into pre-hydrated dialysis membranes (Molecular-weight cutoff [MWCO] 8000–12,000 Daltons) and immersed in 100 mL of phosphate-buffered saline (PBS, 0.05 M, pH 7.4) at 37 °C under constant agitation (100 rpm). Aliquots were collected at predetermined intervals (0.5–24 h) and replaced with fresh PBS to maintain sink conditions. Free quercetin, free resveratrol, and an unencapsulated 50:50 quercetin–resveratrol mixture were prepared from individual DMSO (10%) stock solutions diluted in phosphate-buffered saline (PBS; 0.05 M, pH 7.4). These controls did not contain niosomes. For each control, 10 mL of solution was placed in a pre-hydrated dialysis membrane and tested under the same receiving-medium, temperature, agitation, and sampling conditions as the QR-NIO dispersion.

Released polyphenols were quantified by ultraviolet–visible (UV–Vis) spectrophotometry using calibration curves, and cumulative release was expressed as the percentage of quercetin and resveratrol released over time. Release data were fitted to the Korsmeyer–Peppas equation, Mt/M∞ = kt^n^, where Mt/M∞ represents the released fraction at time t. The fit included only data points within the initial release region (Mt/M∞ ≤ 0.60), excluding the 0 h value. Release data for each time point are provided in Table S1, while the fitted rate constant (k), release exponent (n), and coefficient of determination (R^2^) are reported in Table S2.

### 4.7. Preparation and Evaluation of QR-NIO-Enriched Hummus

#### 4.7.1. Hummus Preparation

Functional hummus was prepared using cooked chickpeas as the principal food matrix, with modifications from previously reported hummus formulations [71,72]. Cooked chickpeas, tahini, extra virgin olive oil, water, lemon juice, sodium chloride, and paprika were combined at the proportions specified in Table 10 and homogenized using a Waring Commercial Laboratory Blender (Model 7011HS, Waring Products, Stamford, CT, USA) at 18,000 rpm for 1 min until a smooth and uniform paste was obtained. The resulting base formulation was divided into equal portions. For the preparation of QR-NIO-enriched functional hummus (QRH), 2.0 g of the selected QR-NIO dispersion was incorporated per 100 g of product, replacing an equivalent amount of water.

The total polyphenol content was standardized to 100 µmol per 100 g of hummus. Based on molecular weights of 302.24 and 228.24 g/mol for quercetin and resveratrol, respectively, the 100:0 and 0:100 endpoints corresponded to 30.22 mg of quercetin and 22.82 mg of resveratrol per 100 g. Accordingly, the selected 50:50 molar formulation provided 15.11 mg of quercetin and 11.41 mg of resveratrol per 100 g of QRH. Intermediate compositions were calculated proportionally according to their respective molar fractions. After QR-NIO incorporation, QRH was mechanically mixed at 500 rpm for 5 min to promote uniform dispersion throughout the food matrix. Non-functionalized hummus (NFH), prepared without QR-NIOs, was processed under otherwise identical conditions. Commercial hummus (CH) was included as an external comparator where indicated. All formulations were aseptically portioned into sterile, airtight containers and stored at 4 ± 1 °C for up to 28 days before physicochemical, microbiological, sensory, antioxidant, and biological evaluations.

#### 4.7.2. Microbiological Analysis

The microbiological stability of functional chickpea hummus enriched with synergistic dual-loaded quercetin–resveratrol niosomes (QR-NIOs) was evaluated over 28 days of refrigerated storage (4 ± 1 °C) at 0, 7, 14, 21, and 28 days [72]. Three independent production batches were prepared, and six biological replicates per batch (*n* = 6) were analyzed at each sampling point, yielding 18 samples per storage time. For microbiological analysis, 25 g of each sample was aseptically homogenized with 225 mL of sterile buffered peptone water, followed by serial decimal dilutions. Results were expressed as colony-forming units per gram (CFU/g) or as presence/absence in 25 g for pathogenic microorganisms. Microbiological quality was assessed by enumerating aerobic mesophilic bacteria, yeasts and molds, total and fecal coliforms, and Enterobacteriaceae, while *Salmonella* spp., *Listeria monocytogenes*, *Staphylococcus aureus*, *Bacillus cereus*, and *Clostridium perfringens* were detected using standardized selective culture and microbiological confirmation procedures.

All microbiological analyses were performed following the standardized analytical procedures established in NOM-210-SSA1-2014 for the detection and enumeration of foodborne microorganisms and in accordance with the hygienic manufacturing criteria described in NOM-251-SSA1-2009. The microbiological quality of the QR-NIO-enriched chickpea hummus throughout refrigerated storage was interpreted according to the corresponding Mexican microbiological specifications applicable to ready-to-eat foods.

Microbiological analyses were performed using the applicable detection and enumeration methods described in NOM-210-SSA1-2014 [73]. Sample preparation and handling followed the relevant hygienic practices described in NOM-251-SSA1-2009 [74]. Acceptance limits and microbiological thresholds were evaluated according to NOM-093-SSA1-1994 [75]. Results for each microorganism were reported across the 28-day refrigerated storage period.

#### 4.7.3. Simulated Gastrointestinal Digestion and Analysis of Its Fractions

The gastrointestinal stability and bioaccessibility of the functional hummus enriched with the selected synergistic dual-loaded quercetin–resveratrol niosomes (QR-NIOs) were evaluated using a static in vitro digestion model comprising oral, gastric, and intestinal phases [76,77]. Briefly, 5 g of hummus was mixed with simulated salivary fluid containing α-amylase (150 U/mL, pH 7.0) and incubated at 37 °C for 10 min under gentle agitation to reproduce oral digestion. Subsequently, the pH was adjusted to 2.0, simulated gastric fluid containing porcine pepsin (315 U/mL) was added, and samples were incubated at 37 °C for 2 h to mimic gastric conditions.

Following gastric digestion, the pH was adjusted to 7.0 with sodium bicarbonate and simulated intestinal fluid containing pancreatin (4 mg/mL), lipase (60 U/mL), trypsin (100 U/mL), and bile salts (10 mM) was incorporated. Digestion proceeded for 2 h at 37 °C under continuous agitation. The resulting digesta were transferred into pre-hydrated dialysis membranes (MWCO 12–14 kilodaltons) and immersed in phosphate buffer to separate dialyzable and non-dialyzable fractions. The dialyzable fraction was considered the potentially bioaccessible fraction, whereas the non-dialyzable fraction represented compounds remaining within the intestinal lumen. Bioaccessibility was calculated as the percentage of quercetin and resveratrol recovered in the dialyzable fraction relative to their initial content using the following equation:

(9) $$\mathrm{Bioaccessibility}\left(\%\right)\frac{\mathrm{Polyphenol}\mathrm{content}\mathrm{in}\mathrm{the}\mathrm{dialysate}\mathrm{fraction}}{\mathrm{Initial}\mathrm{Polyphenol}\mathrm{content}}\times 100$$

Aliquots were collected after the oral, gastric, and intestinal digestion stages and stored at −20 °C until analysis. Each fraction was subsequently evaluated for total phenolic and flavonoid contents, antioxidant, anti-inflammatory, and α-amylase and α-glucosidase inhibitory activities. All analyses were performed in triplicate to assess the protective effect of QR-NIO encapsulation during simulated gastrointestinal digestion.

#### 4.7.4. Sensory Evaluation

Sensory acceptance of the hummus formulations was assessed using a hedonic test with 30 untrained volunteers aged 20–45 years from the university community, following previously reported procedures with minor modifications [32,78]. All participants provided informed consent. Each participant evaluated all three formulations: commercial hummus (CH), experimental hummus without niosomes (NFH), and experimental hummus enriched with 5% (*w*/*w*) of the selected 50:50 quercetin–resveratrol niosomal formulation (QRH). Thus, the product was a within-participant factor. Approximately 15 g of each sample was served at room temperature in identical containers labeled with three-digit codes. Presentation order was randomized among participants, and water and unsalted crackers were provided for palate cleansing between samples.

Participants rated appearance, color, aroma, flavor, bitterness, texture, mouthfeel, spreadability, and overall acceptability on a 10-point hedonic scale ranging from 0 (dislike extremely) to 9 (like extremely). Each participant’s score for each formulation and attribute was recorded, preserving the pairing of the three product evaluations. Scores were summarized as means ± standard deviations.

### 4.8. Statistical Analysis

Unless otherwise stated, experiments were performed in triplicate, and results are presented as mean ± standard deviation (SD). Statistical analyses were performed using JMP Pro version 19 (SAS Institute Inc., Cary, NC, USA). Normality and homogeneity of variance were assessed using the Shapiro–Wilk and Levene tests, respectively. Depending on the experimental design, differences were evaluated by one-way or two-way analysis of variance (ANOVA). One-way ANOVA was used for comparisons involving a single factor, including physicochemical, microbiological, sensory, and genotoxicity measurements, as applicable. Two-way ANOVA was used when two factors were evaluated, such as Q:R ratio and presentation, Q:R ratio and blood-group phenotype, or formulation and digestion stage. The factors and comparison set for individual analyses are specified in the corresponding table footnotes and figure legends. When appropriate, ANOVA was followed by Tukey’s multiple-comparison test, with statistical significance set at *p* < 0.05.

Significant pairwise differences are indicated by compact-letter groups. Means sharing at least one letter (e.g., a and *ab*) are not significantly different, whereas means with no letters in common (e.g., a and *b*) differ significantly at *p* < 0.05. Letters are interpreted only within the comparison set defined for each table or figure; identical letters in different assays, panels, or tables do not imply statistical equivalence. Where both lowercase and uppercase letters appear, they denote distinct comparison sets, as specified in the respective footnote or legend. Graphs were generated using GraphPad Prism 10 (GraphPad Software, San Diego, CA, USA).

## 5. Limitations

This study provides an in vitro assessment of quercetin–resveratrol niosomes and their incorporation into hummus. The antioxidant, erythrocyte-protection, and enzyme-inhibition assays do not establish physiological or clinical efficacy. The interaction indices describe deviations from a calculated additive expectation under the tested conditions but do not establish statistically robust synergy across dose–response ranges. Enzyme-inhibition modes and binding sites were not determined. Particle characterization and overall encapsulation efficiency do not confirm that both polyphenols were present in every vesicle, while spectrophotometric measurements in the food and digestion matrices do not identify the individual compounds or their metabolites. Dialysis and simulated digestion indicate apparent release and potentially bioaccessible fractions, not epithelial uptake or systemic bioavailability. Microbiological observations were limited to the tested refrigerated storage conditions and did not include an inoculated challenge study. Finally, sensory acceptance was assessed with untrained participants and does not establish broader consumer acceptance. Compound-specific analytical measurements, transport and metabolism studies, microbiological challenge testing, and appropriately designed in vivo studies are needed to address these limitations.

## 6. Conclusions

This study demonstrates that the functional interactions between quercetin and resveratrol depend on their molar ratio, analytical environment, biological endpoint, and delivery matrix. The equimolar 50:50 formulation provided the broadest favorable response, although the interactions varied among additive, synergistic, and antagonistic effects, precluding claims of universal synergy. Niosomal formulation converted this selected ratio into a nanoscale vesicular system with high overall encapsulation efficiency, initial colloidal stability, and sustained apparent release. Incorporation of QR-NIOs into hummus maintained sensory acceptance comparable to that of commercial hummus and satisfactory microbiological quality during refrigerated storage, supporting its feasibility as a functional-food prototype without demonstrating a specific antimicrobial effect.

Following simulated digestion, QRH fractions retained antioxidant and enzyme-inhibitory activities and showed increased intestinal recovery of assay-reactive phenolic and flavonoid compounds. However, these responses may reflect contributions from quercetin, resveratrol, their transformation products, and compounds released from the chickpea matrix. Therefore, the observed apparent bioaccessibility and in vitro inhibition of PPE, α-amylase, and α-glucosidase demonstrate technological and functional potential but do not establish compound-specific intestinal absorption, systemic bioavailability, disease prevention, or clinical efficacy. QRH may consequently be considered a promising dietary delivery prototype that requires compound-specific chromatographic characterization and further in vitro, in vivo, and clinical validation.

## Figures and Tables

**Table 1:** Erythroprotective activity of QR-NIOs in different ABO/RhD blood groups.

	Inhibition of Hemolysis (%)				
Q:R	A RhD+	B RhD+	AB RhD+	O RhD+	O RhD−
0/100	87.14 ^Bc^ ± 2.53	73.06 ^Dc^ ± 1.35	92.76 ^Ab^ ± 2.42	88.45 ^ABa^ ± 1.67	76.47 ^Cb^ ± 1.35
25/75	83.74 ^Ad^ ± 2.74	64.74 ^Dd^ ± 2.42	76.25 ^Bd^ ± 2.13	82.6 ^ABb^ 3 ± 3.73	71.73 ^Cc^ ± 2.73
50/50	100.00 ^Aa^ ± 1.24	87.54 ^Ba^ ± 2.75	92.64 ^Bb^ ± 1.24	79.13 ^Cb^ ± 2.85	64.73 ^Dd^ ± 2.46
75/25	88.63 ^Ac^ ± 3.63	76.85 ^Cb^ ± 2.49	86.62 ^Ac^ ± 2.35	83.84 ^Bb^ ± 3.74	78.53 ^BCab^ ± 2.63
100/0	94.88 ^Ab^ ± 3.23	72.85 ^Cbc^ ± 2.93	98.53 ^Aa^ ± 2.75	81.96 ^Bb^ ± 2.83	81.87 ^Ba^ ± 2.84

Values are expressed as mean ± standard deviation (SD; *n* = 3) and represent the percentage inhibition of AAPH-induced hemolysis relative to the AAPH-treated control. Data were analyzed by two-way analysis of variance (ANOVA), with quercetin:resveratrol (Q:R) ratio and ABO/RhD blood-group phenotype as factors, followed by Tukey’s multiple-comparison test (*p* < 0.05). Within each blood-group column, values that do not share a lowercase superscript letter differ significantly among Q:R ratios. Within each Q:R row, values that do not share an uppercase superscript letter differ significantly among blood-group phenotypes.

**Table 2:** Experimental and theoretical antioxidant activities of dual-loaded quercetin–resveratrol niosomes (QR-NIOs) determined by the DPPH radical scavenging assay.

Q:R (%)	Experimental (%)	Expected (%)	Index II	Interaction
0:100	49.27	49.27	1.000	Individual
25:75	51.33	51.18	1.003	Additive
50:50	53.41	53.08	1.006	Additive
75:25	51.74	54.99	0.941	Slightly antagonistic
100:0	56.89	56.89	1.000	Individual

The expected inhibitory activity was calculated assuming an additive interaction according to E*_expected_* = F*_q_* − E*_q_*) + (F*_r_* − E*_r_*), where F*_q_* and F*_r_* represent the weight fractions of quercetin and resveratrol, respectively, and E*_q_* and E*_r_* correspond to the inhibitory activities of each compound tested individually at the same total concentration.

**Table 3:** Experimental and theoretical ABTS radical scavenging activities and interaction index of QR-NIOs.

Q:R (%)	Experimental (%)	Expected (%)	Index II	Interaction
0:100	83.61	83.61	1.000	Individual
25:75	73.83	84.29	0.876	Antagonistic
50:50	95.46	84.98	1.123	Synergistic *
75:25	92.76	85.66	1.083	Synergistic *
100:0	86.34	86.34	1.000	Individual

The expected inhibitory activity was calculated assuming an additive interaction according to E*_expected_* = F*_q_* − E*_q_*) + (F*_r_* − E*_r_*), where F*_q_* and F*_r_* represent the weight fractions of quercetin and resveratrol, respectively, and E*_q_* and E*_r_* correspond to the inhibitory activities of each compound tested individually at the same total concentration. The interaction index (II) was calculated as II = E*_experimental_*/E*_expected_*. Interactions were classified as synergistic (II > 1.05), additive (0.95 ≤ II ≤ 1.05), or antagonistic (II < 0.95). An asterisk (*) identifies values classified as synergistic according to these thresholds; it does not denote statistical significance.

**Table 4:** Experimental erythroprotective activity, theoretical additive activity, interaction index (II), and interaction classification of QR-NIOs. Interaction was classified as synergistic (II > 1.00), additive (0.95 ≤ II ≤ 1.05), or antagonistic (II < 0.95).

Q:R	Blood Group	Experimental Activity (%)	Expected Activity (%)	Interaction Index (II)	Interaction
25:75	A RhD+	83.74	89.12	0.940	Antagonistic
50:50	A RhD+	100.45	90.99	1.104 *	Synergistic *
75:25	A RhD+	88.63	92.87	0.954	Additive
25:75	B RhD+	64.74	72.97	0.887	Antagonistic
50:50	B RhD+	87.54	72.96	1.200 *	Synergistic *
75:25	B RhD+	76.85	72.90	1.054 *	Synergistic *
25:75	AB RhD+	76.25	94.20	0.809	Antagonistic
50:50	AB RhD+	92.64	95.65	0.969	Additive
75:25	AB RhD+	86.62	97.09	0.892	Antagonistic
25:75	O RhD+	82.63	86.83	0.952	Additive
50:50	O RhD+	79.13	85.21	0.929	Antagonistic
75:25	O RhD+	83.84	83.58	1.003	Additive
25:75	O RhD−	71.73	77.82	0.922	Antagonistic
50:50	O RhD−	64.73	79.17	0.818	Antagonistic
75:25	O RhD−	78.53	80.52	0.975	Additive

The expected activity was calculated from the weighted contribution of QR-NIOs tested individually at equivalent concentrations. The interaction index (II) was calculated as the ratio between the experimentally observed activity and the theoretical additive activity (II = Eexperimental/Eexpected). The interaction index (II) was calculated as the observed activity divided by the expected additive activity. Interactions were classified as synergistic (II > 1.05), additive (0.95 ≤ II ≤ 1.05), or antagonistic (II < 0.95). An asterisk (*) identifies values classified as synergistic according to these thresholds; it does not denote statistical significance.

**Table 5:** Experimental and theoretical anti-inflammatory activity of dual-loaded quercetin–resveratrol niosomes (QR-NIOs) determined by the porcine pancreatic elastase inhibition (%) assay.

Q:R (%)	PPE Inhibition (%)	Expected (%)	Index II	Interaction
0:100	18.42	18.42	1.000	Individual
25:75	53.56	23.99	2.233	Synergistic *
50:50	57.86	29.56	1.957	Synergistic *
75:25	51.53	35.13	1.467	Synergistic *
100:0	40.70	40.70	1.000	Individual

The expected inhibitory activity was calculated assuming an additive interaction according to E*_expected_* = F*_q_* − E*_q_*) + (F*_r_* − E*_r_*), where F*_q_* and F*_r_* represent the weight fractions of quercetin and resveratrol, respectively, and E*_q_* and E*_r_* correspond to the inhibitory activities of each compound tested individually at the same total concentration. The interaction index (II) was calculated as the observed activity divided by the expected additive activity. Interactions were classified as synergistic (II > 1.05), additive (0.95 ≤ II ≤ 1.05), or antagonistic (II < 0.95). An asterisk (*) identifies values classified as synergistic according to these thresholds; it does not denote statistical significance.

**Table 6:** Experimental and expected α-amylase inhibition (%) of quercetin–resveratrol niosomes (QR-NIOs) at different molar ratios.

Q:R (%)	α-Amylase Inhibition (%)	Expected (%)	Index II	Interaction
0:100	84.57	84.57	1.000	Individual
25:75	82.33	86.24	0.954	Additive
50:50	93.23	87.91	1.061	Synergistic *
75:25	85.24	89.57	0.952	Additive
100:0	91.24	91.24	1.000	Individual

The expected inhibitory activity was calculated assuming an additive interaction according to E*_expected_* = F*_q_* − E*_q_*) + (F*_r_* − E*_r_*), where F*_q_* and F*_r_* represent the weight fractions of quercetin and resveratrol, respectively, and E*_q_* and E*_r_* correspond to the inhibitory activities of each compound tested individually at the same total concentration. The interaction index (II) was calculated as the observed activity divided by the expected additive activity. Interactions were classified as synergistic (II > 1.05), additive (0.95 ≤ II ≤ 1.05), or antagonistic (II < 0.95). An asterisk (*) identifies values classified as synergistic according to these thresholds; it does not denote statistical significance.

**Table 7:** Experimental and expected α-glucosidase inhibition (%) of quercetin–resveratrol niosomes (QR-NIOs) at different molar ratios.

Q:R (%)	α-Glucosidase Inhibition (%)	Expected (%)	Index II	Interaction
0:100	89.34	89.34	1.000	Individual
25:75	79.74	86.69	0.920	Antagonistic
50:50	89.34	84.04	1.063	Synergistic *
75:25	71.42	81.39	0.878	Antagonistic
100:0	78.74	78.74	1.000	Individual

The expected inhibitory activity was calculated assuming an additive interaction according to E*_expected_* = F*_q_* − E*_q_*) + (F*_r_* − E*_r_*), where F*_q_* and F*_r_* represent the weight fractions of quercetin and resveratrol, respectively, and E*_q_* and E*_r_* correspond to the inhibitory activities of each compound tested individually at the same total concentration. The interaction index (II) was calculated as the observed activity divided by the expected additive activity. Interactions were classified as synergistic (II > 1.05), additive (0.95 ≤ II ≤ 1.05), or antagonistic (II < 0.95). An asterisk (*) identifies values classified as synergistic according to these thresholds; it does not denote statistical significance.

**Table 8:** Antioxidant capacity of CH, NFH, and QRH throughout simulated gastrointestinal digestion.

		Inhibition (%)			µmol TE/g D.W.	
		DPPH•	ABTS•+	AAPH	FRAP	DMPD•+
CH	OP	34.79 ^e^ ± 2.48	32.06 ^f^ ± 3.63	3.63 ^h^ ± 2.74	975.64 ^d^ ± 41.75	871.43 ^f^ ± 152.86
	GP	30.06 ^ef^ ± 3.35	70.53 ^c^± 3.93	11.73 ^g^ ± 3.37	739.33 ^e^ ± 22.13	1062.69 ^e^ ± 123.25
	DIP	75.45 ^d^ ± 2.74	73.07 ^c^ ± 2.52	23.73 ^f^ ± 1.41	1079.05 ^cd^ ± 24.43	1270.48 ^d^ ± 98.75
	NDP	88.69 ^b^ ± 1.76	70.48 ^cd^ ± 2.68	54.26 ^d^ ± 3.36	1261.10 ^cd^ ± 74.14	1329.14 ^d^ ± 312.14
NFH	OP	29.79 ^f^ ± 2.73	59.87 ^e^ ± 1.54	14.74 ^d^ ± 2.62	1142.65 ^cd^ ± 84.65	845.71 ^f^ ± 223.44
	GP	74.04 ^d^ ± 3.74	65.25 ^d^ ± 2.57	28.46 ^e^ ± 3.86	963.23 ^d^ ± 83.75	1034.75 ^d^ ± 125.52
	DIP	88.45 ^b^ ± 4.62	83.53 ^b^ ± 1.24	65.73 ^c^ ± 1.48	1167.90 ^cd^ ± 103.86	1741.48 ^cd^ ± 123.72
	NDP	98.30 ^a^ ± 2.96	91.84 ^a^ ± 2.47	76.84 ^b^ ± 3.35	2530.96 ^b^ ± 135.43	2352.14 ^b^ ± 234.73
QRH	OP	31.36 ^e^ ± 1.63	68.34 ^d^ ± 3.83	12.66 ^g^ ± 4.25	1350.75 ^c^ ± 186.86	1842.86 ^c^ ± 153.23
	GP	24.17 ^f^ ± 3.53	73.42 ^d^ ± 3.16	25.53 ^ef^ ± 1.87	1320.24 ^c^ ± 175.46	1366.67 ^de^ ± 223.41
	DIP	82.22 ^c^ ± 2.42	85.64 ^b^ ± 2.86	76.26 ^b^ ± 2.35	2304.76 ^b^ ± 87.35	2576.19 ^ab^ ± 126.71
	NDP	92.18 ^ab^ ± 3.63	93.64 ^a^ ± 1.24	89.63 ^a^ ± 2.62	4233.41 ^a^ ± 256.24	2742.86 ^a^ ± 203.15

Values are presented as mean ± standard deviation. Within each antioxidant assay column, lowercase superscript letters compare the 12 combinations of hummus formulation and digestion stage or intestinal fraction. Values sharing at least one letter do not differ significantly; values with no letters in common differ significantly (*p* < 0.05). Letters must not be compared across different assays. DPPH•, ABTS•+, and AAPH results are expressed as inhibition (%); FRAP and DMPD•+ results are expressed as micromoles of Trolox equivalents per gram of dry weight (µmol TE/g D.W.). OP, oral phase; GP, gastric phase; DIP, dialyzable intestinal fraction; NDP, non-dialyzable intestinal fraction. CH, commercial hummus; NFH, non-functionalized hummus; QRH, QR-NIO-enriched hummus.

**Table 9:** Sensory evaluation of QR-NIO-enriched functional hummus.

Sensory Attributes	Treatments			
	CH	NFH	QRH	Product Effect (*p*-Value)
Appearance	7.24 ^b^ ± 0.32	7.41 ^b^ ± 0.21	8.35 ^a^ ± 0.27	<0.001
Color	6.43 ^b^ ± 0.44	6.64 ^b^ ± 0.32	7.76 ^a^ ± 0.67	<0.001
Aroma	8.63 ^a^ ± 0.26	8.24 ^b^ ± 0.25	8.67 ^a^ ± 1.13	<0.001
Flavor	8.35 ^ab^ ± 0.32	8.16 ^b^ ± 0.45	8.47 ^a^ ± 0.42	0.0072
Bitterness	8.29 ^a^ ± 0.25	7.78 ^b^ ± 0.23	8.74 ^a^ ± 0.28	<0.001
Texture	8.19 ^a^ ± 0.40	7.81 ^b^ ± 0.52	8.10 ^ab^ ± 0.45	0.0471
Mouthfeel	7.43 ^a^ ± 0.15	6.76 ^b^ ± 0.21	7.56 ^a^ ± 0.33	<0.001
Spreadability	7.66 ^a^ ± 0.12	6.91 ^b^ ± 0.20	7.37 ^b^ ± 0.27	<0.001
Overall Acceptability	8.32 ^a^ ± 0.23	7.83 ^b^ ± 0.42	8.22 ^a^ ± 0.83	0.0026

Values are means ± standard deviations from 30 panelists, each of whom evaluated all three hummus formulations. Each sensory attribute was analyzed separately using a linear mixed-effects model with formulation as a fixed effect and panelist as a random effect. Pairwise comparisons of formulations were adjusted using Tukey’s method. Within each row, means sharing a letter do not differ significantly (*p* ≥ 0.05). The *p*-value in the final column corresponds to the overall effect of the formulation. CH, commercial hummus; NFH, non-functionalized hummus; QRH, QR-NIO-enriched functional hummus.

**Table 10:** Formulation of hummus per 100 g of product.

Ingredients	Amount (g/100 g)
Cooked and mashed chickpeas	70.0
Tahini	10.0
Extra virgin olive oil	7.0
Water	6.8
Lemon juice	5.0
Sodium chloride	1.0
Paprika	0.2
Total	100.0

For QRH preparation, the QR-NIO dispersion replaced an equivalent amount of water to maintain a final product mass of 100 g.

## Data Availability

All data supporting the conclusions of this study are included in the article. Further information or clarification may be requested from the corresponding authors, provided that the request is reasonable.

## Supplementary Materials

The following supporting information can be downloaded at: https://www.mdpi.com/article/10.3390/ijms27198686/s1.

## Abbreviations

The following abbreviations are used in this manuscript:

AAPH	2,2′-Azobis(2-methylpropionamidine) dihydrochloride
ABO	Human blood groups
ABTS	2,2′-Azinobis(3-ethylbenzothiazoline-6-sulfonic acid)
AMPK	AMP-activated protein kinase
ANOVA	Analysis of variance
CFU	Colony-forming units
CH	Commercial hummus
DIP	Dialyzable intestinal phase
DLS	Dynamic light scattering
DMPD	*N*,*N*-Dimethyl-p-phenylenediamine
DMSO	Dimethyl sulfoxide
DPPH	2,2-Diphenyl-1-picrylhydrazyl
D.W.	Dry weight
EDTA	Ethylenediaminetetraacetic acid
FRAP	Ferric reducing antioxidant power
GAE	Gallic acid equivalents
GP	Gastric phase
LC–MS	Liquid chromatography–mass spectrometry
II	Interaction index
MWCO	Molecular-weight cutoff
NCDs	Non-communicable diseases
NDP	Non-dialyzable intestinal phase
NFH	Non-functionalized hummus
NF-κB	Nuclear factor kappa B
NLRP3	NOD-like receptor family pyrin domain-containing 3
Nrf2	Nuclear factor erythroid 2-related factor 2
OP	Oral phase
QE	Quercetin equivalents
PBS	Phosphate-buffered saline
PDI	Polydispersity index
PPE	Porcine pancreatic elastase
Q	Quercetin-to-resveratrol ratio
QRH	Quercetin–resveratrol niosome-enriched hummus
QR-NIOs	Dual-loaded quercetin–resveratrol niosomes
Q–R	Quercetin–resveratrol
QUE	Quercetin
RES	Resveratrol
RhD	Rhesus D antigen
SD	Standard deviation
SEM	Scanning electron microscopy
TE	Trolox equivalents
UV–Vis	Ultraviolet–visible

## References

[1] World Health Organization Noncommunicable Diseases. https://www.who.int/news-room/fact-sheets/detail/noncommunicable-diseases.

[2] (2024). GBD 2021 Causes of Death Collaborators. Global burden of 288 causes of death and life expectancy decomposition in 204 countries and territories and 811 subnational locations, 1990–2021: A systematic analysis for the Global Burden of Disease Study 2021. Lancet.

[3] Cani P.D., Amar J., Iglesias M.A., Poggi M., Knauf C., Bastelica D., Neyrinck A.M., Fava F., Tuohy K.M., Chabo C. (2007). Metabolic endotoxemia initiates obesity and insulin resistance. Diabetes.

[4] Vandanmagsar B., Youm Y.H., Ravussin A., Galgani J.E., Stadler K., Mynatt R.L., Ravussin E., Stephens J.M., Dixit V.D. (2011). The NLRP3 inflammasome instigates obesity-induced inflammation and insulin resistance. Nat. Med..

[5] Uruno A., Furusawa Y., Yagishita Y., Fukutomi T., Muramatsu H., Negishi T., Sugawara A., Kensler T.W., Yamamoto M. (2013). The Keap1–Nrf2 system prevents onset of diabetes mellitus. Mol. Cell. Biol..

[6] Cani P.D., Bibiloni R., Knauf C., Waget A., Neyrinck A.M., Delzenne N.M., Burcelin R. (2008). Changes in gut microbiota control metabolic endotoxemia-induced inflammation in high-fat diet-induced obesity and diabetes in mice. Diabetes.

[7] Braniste V., Al-Asmakh M., Kowal C., Anuar F., Abbaspour A., Tóth M., Korecka A., Bakocevic N., Ng L.G., Kundu P. (2014). The gut microbiota influences blood–brain barrier permeability in mice. Sci. Transl. Med..

[8] Erny D., Hrabě de Angelis A.L., Jaitin D., Wieghofer P., Staszewski O., David E., Keren-Shaul H., Mahlakoiv T., Jakobshagen K., Buch T. (2015). Host microbiota constantly control maturation and function of microglia in the CNS. Nat. Neurosci..

[9] Etxeberria U., Arias N., Boqué N., Macarulla M.T., Portillo M.P., Martínez J.A., Milagro F.I. (2015). Reshaping faecal gut microbiota composition by the intake of trans-resveratrol and quercetin in high-fat sucrose diet-fed rats. J. Nutr. Biochem..

[10] Zhao L., Zhang Q., Ma W., Tian F., Shen H., Zhou M. (2017). A combination of quercetin and resveratrol reduces obesity in high-fat diet-fed rats by modulation of gut microbiota. Food Funct..

[11] Suchankova G., Nelson L.E., Gerhart-Hines Z., Kelly M., Gauthier M.S., Saha A.K., Ido Y., Puigserver P., Ruderman N.B. (2009). Concurrent regulation of AMP-activated protein kinase and SIRT1 in mammalian cells. Biochem. Biophys. Res. Commun..

[12] Csiszar A., Labinskyy N., Pinto J.T., Ballabh P., Zhang H., Losonczy G., Pearson K., de Cabo R., Pacher P., Zhang C. (2009). Resveratrol induces mitochondrial biogenesis in endothelial cells. Am. J. Physiol. Heart Circ. Physiol..

[13] Walle T., Hsieh F., DeLegge M.H., Oatis J.E., Walle U.K. (2004). High absorption but very low bioavailability of oral resveratrol in humans. Drug Metab. Dispos..

[14] Lee S.Y., Lee S.J., Yim D.G., Hur S.J. (2020). Changes in the content and bioavailability of onion quercetin and grape resveratrol during in vitro human digestion. Foods.

[15] Pando D., Beltrán M., Gerone I., Matos M., Pazos C. (2015). Resveratrol entrapped niosomes as yoghurt additive. Food Chem..

[16] Schlich M., Lai F., Pireddu R., Pini E., Ailuno G., Fadda A.M., Valenti D., Sinico C. (2020). Resveratrol proniosomes as a convenient nanoingredient for functional food. Food Chem..

[17] Yang Z., McClements D.J., Peng X., Xu Z., Meng M., Chen L., Jin Z. (2023). Fabrication of zein–carboxymethyl cellulose nanoparticles for co-delivery of quercetin and resveratrol. J. Food Eng..

[18] Yang Z., McClements D.J., Peng X., Qiu C., Long J., Zhao J., Xu Z., Meng M., Chen L., Jin Z. (2022). Co-encapsulation of quercetin and resveratrol in zein/carboxymethyl cellulose nanoparticles: Characterization, stability and in vitro digestion. Food Funct..

[19] Alvarez M.D., Fuentes R., Guerrero G., Canet W. (2017). Characterization of commercial Spanish hummus formulation: Nutritional composition, rheology, and structure. Int. J. Food Prop..

[20] O’Neil C.E., Nicklas T.A., Fulgoni V.L. (2014). Chickpeas and hummus are associated with better nutrient intake, diet quality, and levels of some cardiovascular risk factors: National Health and Nutrition Examination Survey 2003–2010. J. Nutr. Food Sci..

[21] Frankenfeld C.L., Wallace T.C. (2020). Dietary patterns and nutritional status in relation to consumption of chickpeas and hummus in the U.S. population. Appl. Sci..

[22] Lu B., Huang Y., Chen Z., Ye J., Xu H., Chen W., Long X. (2019). Niosomal Nanocarriers for Enhanced Skin Delivery of Quercetin with Functions of Anti-Tyrosinase and Antioxidant. Molecules.

[23] Machado N.D., Gutiérrez G., Matos M., Fernández M.A. (2021). Preservation of the Antioxidant Capacity of Resveratrol via Encapsulation in Niosomes. Foods.

[24] González-Vega R.I., Robles-García M.Á., Mendoza-Urizabel L.Y., Cárdenas-Enríquez K.N., Ruiz-Cruz S., Gutiérrez-Lomelí M., Iturralde-García R.D., Avila-Novoa M.G., Villalpando-Vargas F.V., Del-Toro-Sánchez C.L. (2023). Impact of the ABO and RhD Blood Groups on the Evaluation of the Erythroprotective Potential of Fucoxanthin, β-Carotene, Gallic Acid, Quercetin and Ascorbic Acid as Therapeutic Agents against Oxidative Stress. Antioxidants.

[25] Chen Y., Deuster P. (2009). Comparison of Quercetin and Dihydroquercetin: Antioxidant-Independent Actions on Erythrocyte and Platelet Membrane. Chem. Biol. Interact..

[26] Brás N.F., Gonçalves R., Mateus N., Fernandes P.A., Ramos M.J., de Freitas V. (2010). Inhibition of Pancreatic Elastase by Polyphenolic Compounds. J. Agric. Food Chem..

[27] Lin L., Yao H., Fu J., Zhang W., Li Y., Wang Y., Shen H., Mo J., Guan Y. (2025). Elastase Inhibition by Natural Flavonoids: Mechanistic Insights and Potential Therapeutic Applications. Front. Nutr..

[28] Wang L., Ma R., Tian Y. (2024). Quercetin Slow-Release System Delays Starch Digestion via Inhibiting Transporters and Enzymes. Food Chem..

[29] Zhang A.J., Rimando A.M., Mizuno C.S., Mathews S.T. (2017). α-Glucosidase Inhibitory Effect of Resveratrol and Piceatannol. J. Nutr. Biochem..

[30] Javani R., Hashemi F.S., Ghanbarzadeh B., Hamishehkar H. (2021). Quercetin-Loaded Niosomal Nanoparticles Prepared by the Thin-Layer Hydration Method: Formulation Development, Colloidal Stability, and Structural Properties. LWT-Food Sci. Technol..

[31] Ritger P.L., Peppas N.A. (1987). A Simple Equation for Description of Solute Release I: Fickian and Non-Fickian Release from Non-Swellable Devices in the Form of Slabs, Spheres, Cylinders or Discs. J. Control. Release.

[32] Karam L., Ghonim F., Dahdah P., Attieh G., Al-Ahmad S., Ghonim S., Osaili T. (2023). Beyond Chemical Preservatives: Enhancing the Shelf-Life and Sensory Quality of Ready-to-Eat (RTE) Hummus with Vinegar and Other Natural Antimicrobials. Foods.

[33] Perez-Perez L.M., Huerta-Ocampo J.A., Ruiz-Cruz S., Cinco-Moroyoqui F.J., Rosas-Burgos E.C., Corella-Madueño M.A.G., Del-Toro-Sánchez C.L. (2021). Evaluation of Quality, Antioxidant Capacity, and Digestibility of Chickpea (*Cicer arietinum* L. cv. Blanoro) Stored under N_2_ and CO_2_ Atmospheres. Molecules.

[34] Liu Y., Liang X., Zou Y., Peng Y., McClements D.J., Hu K. (2020). Resveratrol-Loaded Biopolymer Core–Shell Nanoparticles: Bioavailability and Anti-Inflammatory Effects. Food Funct..

[35] Kurin E., Mučaji P., Nagy M. (2012). In vitro antioxidant activities of three red wine polyphenols and their mixtures: An interaction study. Molecules.

[36] Giordano M.E., Lionetto M.G. (2023). Intracellular redox behavior of quercetin and resveratrol singly and in mixtures. Molecules.

[37] Hapner C.D., Deuster P., Chen Y. (2010). Inhibition of oxidative hemolysis by quercetin, but not other antioxidants. Chem.-Biol. Interact..

[38] Tadera K., Minami Y., Takamatsu K., Matsuoka T. (2006). Inhibition of α-glucosidase and α-amylase by flavonoids. J. Nutr. Sci. Vitaminol..

[39] Shen Y., Xu Z., Sheng Z. (2017). Ability of Resveratrol to Inhibit Advanced Glycation End Product Formation and Carbohydrate-Hydrolyzing Enzyme Activity, and to Conjugate Methylglyoxal. Food Chem..

[40] Martinez-Gonzalez A.I., Díaz-Sánchez Á.G., de la Rosa L.A., Bustos-Jaimes I., Alvarez-Parrilla E. (2019). Inhibition of α-Amylase by Flavonoids: Structure–Activity Relationship. Spectrochim. Acta A Mol. Biomol. Spectrosc..

[41] Li Y.Q., Zhou F.C., Gao F., Bian J.S., Shan F. (2009). Comparative Evaluation of Quercetin, Isoquercetin and Rutin as Inhibitors of α-Glucosidase. J. Agric. Food Chem..

[42] Elmowafy E., El-Derany M.O., Biondo F., Tiboni M., Casettari L., Soliman M.E. (2020). Quercetin Loaded Monolaurate Sugar Esters-Based Niosomes: Sustained Release and Mutual Antioxidant–Hepatoprotective Interplay. Pharmaceutics.

[43] Ritwiset A., Krongsuk S., Johns J.R. (2016). Molecular Structure and Dynamical Properties of Niosome Bilayers with and without Cholesterol Incorporation: A Molecular Dynamics Simulation Study. Appl. Surf. Sci..

[44] Nematollahi M.H., Pardakhty A., Torkzadeh-Mahanai M., Mehrabani M., Asadikaram G. (2017). Changes in Physical and Chemical Properties of Niosome Membrane Induced by Cholesterol: A Promising Approach for Niosome Bilayer Intervention. RSC Adv..

[45] Zheng B., Zhou H., McClements D.J. (2023). Co-Encapsulation of Multiple Polyphenols in Plant-Based Milks: Formulation, Gastrointestinal Stability, and Bioaccessibility. Foods.

[46] Milán-Noris A.K., Gutiérrez-Uribe J.A., Santacruz A., Serna-Saldívar S.O., Martínez-Villaluenga C. (2018). Peptides and Isoflavones in Gastrointestinal Digests Contribute to the Anti-Inflammatory Potential of Cooked or Germinated Desi and Kabuli Chickpea. Food Chem..

[47] De Santi C., Pietrabissa A., Mosca F., Pacifici G.M. (2000). Glucuronidation of Resveratrol, a Natural Product Present in Grape and Wine, in the Human Liver. Xenobiotica.

[48] Jaisamut P., Wanna S., Limsuwan S., Chusri S., Wiwattanawongsa K., Wiwattanapatapee R. (2021). Enhanced Oral Bioavailability and Improved Biological Activities of a Quercetin/Resveratrol Combination Using a Liquid Self-Microemulsifying Drug Delivery System. Planta Med..

[49] (2012). For the Disposal of Human Blood and Its Components for Therapeutic Pu Poses.

[50] (2022). General Requirements for the Competence of Testing and Calibration Laboratories.

[51] (2022). Medical Laboratories—Requirements for Quality and Competence.

[52] (2022). Clinical Laboratory Testing and In Vitro Diagnostic Test Systems.

[53] (2015). Quality Management Systems.

[54] Brand-Williams W., Cuvelier M.E., Berset C. (1995). Use of a Free Radical Method to Evaluate Antioxidant Activity. LWT-Food Sci. Technol..

[55] Re R., Pellegrini N., Proteggente A., Pannala A., Yang M., Rice-Evans C. (1999). Antioxidant Activity Applying an Improved ABTS Radical Cation Decolorization Assay. Free Radic. Biol. Med..

[56] Benzie I.F.F., Strain J.J. (1996). The Ferric Reducing Ability of Plasma (FRAP) as a Measure of “Antioxidant Power”: The FRAP Assay. Anal. Biochem..

[57] Fogliano V., Verde V., Randazzo G., Ritieni A. (1999). Method for Measuring Antioxidant Activity and Its Application to Monitoring the Antioxidant Capacity of Wines. J. Agric. Food Chem..

[58] Thabet Y., Elsabahy M., Eissa N.G. (2022). Methods for Preparation of Niosomes: A Focus on the Thin-Film Hydration Method. Methods.

[59] Zanganeh E., Mirzaei H., Jafari S.M., Javadi A., Afshar Mogaddam M.R. (2022). Spirulina platensis Extract Nanoliposomes: Preparation, Characterization, and Application to White Cheese. J. AOAC Int..

[60] Ruiz-Cruz S., González-Vega R.I., Robles-Zepeda R.E., Reyes-Díaz A., López-Elías J.A., Alvarez-Ainza M.L., Cinco-Moroyoqui F.J., Moreno-Corral R.A., Wong-Corral F.J., Borboa-Flores J. (2022). Association of Different ABO and Rh Blood Groups with the Erythroprotective Effect of Extracts from Navicula incerta and Their Anti-Inflammatory and Antiproliferative Properties. Metabolites.

[61] Lee K.K., Kim J.H., Cho J.J., Choi J.D. (1999). Inhibitory Effects of 150 Plant Extracts on Elastase Activity, and Their Anti-Inflammatory Effects. Int. J. Cosmet. Sci..

[62] Stojanova M., Stojanova M.S.T., Djukic D.A., Popovska O., Sabriu Haxhijaha A., Kaya Y. (2026). Protein–β-Glucan Nanoparticle-Enriched Peach Juice: In Vitro Bioaccessibility, Metabolic Enzyme Inhibition, and Sensory Evaluation. Foods.

[63] Wang J., Zhao M., Liu T., Feng F., Zhou A. (2022). Guidelines for the Digestive Enzymes Inhibition Assay. eFood.

[64] Gościniak A., Szulc P., Zielewicz W., Walkowiak J., Cielecka-Piontek J. (2023). Multidirectional Effects of Red Clover (*Trifolium pratense* L.) in Support of Menopause Therapy. Molecules.

[65] Wang S., Meckling K.A., Marcone M.F., Kakuda Y., Tsao R. (2011). Synergistic, Additive, and Antagonistic Effects of Food Mixtures on Total Antioxidant Capacities. J. Agric. Food Chem..

[66] Sadowska-Bartosz I., Bartosz G. (2026). Interactions between Antioxidants: How Useful Are In Vitro Studies for Understanding the Action of Antioxidants in Complex Systems?. Int. J. Mol. Sci..

[67] Xu Y., Guo Y., Yang Y., Meng Y., Xia X., Liu Y. (2019). Stabilization of Deformable Nanovesicles Based on Insulin–Phospholipid Complex by Freeze-Drying. Pharmaceutics.

[68] Turkez H., Oner S., Yıldırım O.C., Arslan M.E., Dimmito M.P., Kahraman Ç.Y., Marinelli L., Sonmez E., Kiki Ö., Tatar A. (2025). Synthesis and Characterization of Memantine-Loaded Niosomes for Enhanced Alzheimer’s Disease Targeting. Pharmaceutics.

[69] Kostevšek N., Cheung C.C.L., Serša I., Kreft M.E., Monaco I., Comes Franchini M., Vidmar J., Al-Jamal W.T. (2020). Magneto-liposomes as MRI contrast agents: A systematic study of different liposomal formulations. Nanomaterials.

[70] Tan C., Xue J., Lou X., Abbas S., Guan Y., Feng B., Zhang X., Xia S. (2014). Liposomes as Delivery Systems for Carotenoids: Comparative Studies of Loading Ability, Storage Stability and In Vitro Release. Food Funct..

[71] Doumani N., Severin I., Dahbi L., Bou-Maroun E., Tueni M., Sok N., Chagnon M.-C., Maalouly J., Cayot P. (2020). Lemon Juice, Sesame Paste, and Autoclaving Influence Iron Bioavailability of Hummus: Assessment by an In Vitro Digestion/Caco-2 Cell Model. Foods.

[72] Salazar J.K., Natarajan V., Stewart D., Fay M., Gonsalves L.J., Mhetras T., Sule C., Tortorello M.L. (2020). *Listeria monocytogenes* Growth Kinetics in Refrigerated Ready-to-Eat Dips and Dip Components. PLoS ONE.

[73] (2015). Productos y Servicios. Métodos de Prueba Microbiológicos. Determinación de Microorganismos por Cuenta en Placa, Coliformes Totales, Coliformes Fecales, *Escherichia coli*, *Salmonella* spp., *Listeria monocytogenes*, *Staphylococcus aureus*, *Clostridium perfringens* y *Vibrio cholerae*.

[74] (2010). Prácticas de Higiene para el Proceso de Alimentos, Bebidas o Suplementos Alimenticios.

[75] (1995). Bienes y Servicios. Prácticas de Higiene y Sanidad en la Preparación de Alimentos que se Ofrecen en Establecimientos Fijos.

[76] Van-Campen D.R., Glahn R.P. (1999). Micronutrient bioavailability techniques: Accuracy, problems and limitations. Field Crops Res..

[77] Tarko T., DudaChodak A., Sroka P., Satora P., Michalik J. (2009). Tranformations of phenolic compounds in an in vitro model simulating the human alimentary tract. Food Technol. Biotechnol..

[78] Osaili T.M., Dhanasekaran D.K., Hasan F., Obaid R.S., Al-Nabulsi A.A., Olaimat A.N., Ismail L.C., Hasan H., Ayyash M., Bamigbade G.B. (2025). High Pressure Processing of Hummus: Enhancing Microbial Safety and Stability, and Reducing Lipid Oxidation. Heliyon.

