# Supplementary material for: Dual-Loaded Quercetin–Resveratrol Niosomes: Sustained In Vitro Release and Retained Bioactivity in Hummus During Simulated Gastrointestinal Digestion
Source: Int J Mol Sci. 2026 Sep 28;27(19):8686. doi: 10.3390/ijms27198686 (PMC13665077; doi:10.3390/ijms27198686)
Supplement: Supplementary file 1 [file ijms-27-08686-s001.zip › ijms-4574641-supplementary.pdf]

Supplementary Materials

Tables S1 and S2 provide the numerical data supporting the apparent release profiles and Korsmeyer–Peppas fits shown in Figure 8. Table S1 reports cumulative apparent release at each sampling time as mean ± standard deviation for free quercetin, free resveratrol, their unencapsulated 50:50 mixture, and the corresponding niosomal formulation. Table S2 reports the time points and release interval used for each fit, together with the resulting model parameters. Experimental procedures are described in Section 4.4.3 of the main manuscript.

Table S1. Cumulative release (%) at each sampling time for free quercetin (QUE), free resveratrol (RES), the unencapsulated 50:50 quercetin–resveratrol mixture (Q–R), and quercetin–resveratrol niosomes (QR-NIOs).

Time (h)	QUE	RES	Q-R	QR-NIOs
0	0.00 ±0.00	0.00 ±0.00	0.00 ±0.00	0.00 ±0.00
1	45.69 ±1.24	32.06 ±1.85	21.60 ±1.35	3.38 ±0.65
2	64.11 ±1.86	48.84 ±2.43	34.84 ±2.24	9.86 ±2.73
4	77.55 ±2.75	57.51 ±1.80	44.64 ±0.13	18.62 ±1.46
6	83.21 ±0.57	63.85 ±1.73	52.75 ±2.83	24.38 ±1.46
8	89.26 ±1.96	69.26 ±0.96	59.75 ±0.76	31.81 ±2.76
10	92.14 ±3.21	75.11 ±2.97	63.74 ±2.45	36.57 ±2.24
12	94.97 ±2.03	76.38 ±1.57	67.64 ±0.54	41.66 ±1.85
18	96.81 ±1.63	82.78 ±0.86	73.63 ±0.87	48.58 ±2.24
24	97.52 ±1.74	86.74 ±1.94	80.35 ±1.75	51.63 ±0.86

Values are mean ± standard deviation; specify the number of independent determinations (n) here. QUE, free quercetin; RES, free resveratrol; Q–R, unencapsulated quercetin mixture (50:50, w/w); QR-NIOs, quercetin–resveratrol niosomal formulation (50:50, w/w). The 0-h values define the initial baseline. Percentages describe apparent passage through the dialysis membrane, not gastrointestinal absorption.

Table S2. Korsmeyer–Peppas fit parameters for the apparent release profiles of free quercetin (QUE), free resveratrol (RES), their unencapsulated 50:50 mixture (Q–R), and quercetin–resveratrol niosomes (QR-NIOs).

Sample	k	n	R^2
QUE	0.241	0.523	0.984
RES	0.248	0.425	0.986
Q–R (50:50)	0.194	0.401	0.979
QR-NIOs (50:50)	0.082	0.318	0.991

The table reports the parameters were obtained from the Korsmeyer–Peppas equation, $M_t/M_\infty = kt^n$, where M_t/M_∞ is the released fraction at time t (1-24 h), k is the fitted constant, n is the release exponent, and R^2 is the coefficient of determination. Time zero was excluded because its logarithm is undefined. The parameters describe apparent release through the dialysis membrane and do not, by themselves, establish a vesicle-bilayer transport mechanism.
